# Comparing the effectiveness of prophylactic strategies for parastomal hernia prevention: a network meta-analysis

**DOI:** 10.1007/s10151-025-03211-6

**Published:** 2025-09-25

**Authors:** J. Martín-Arévalo, V. A. López-Callejon, D. Moro-Valdezate, L. Pérez-Santiago, F. López-Mozos, J. A. Carbonell Asins, D. Casado Rodrigo, S. García-Botello, J. Puente Monserrat, V. Pla-Martí

**Affiliations:** 1 Colorectal Surgery Unit, Department of General and Digestive Surgery, Hospital Clínico Universitario de Valencia, Valencia, Spain; 2 Department of Colorectal Surgery, University Clinic Hospital of Valencia, University of Valencia, Av. Blasco Ibáñez, 17, 46010 Valencia, Spain; 3 Stomatherapy Unit, Hospital Clínico Universitario de Valencia, Valencia, Spain; 4 Unit of Biostatistics, INCLIVA Biomedical Research Institute, Valencia, Spain

**Keywords:** Parastomal hernia, Prophylaxis, Meta-analysis, Mesh, Exercises, Abdominal rehabilitation

## Abstract

**Background:**

Parastomal hernia (PSH), a common ostomy complication, significantly impairs patient quality of life. Various prophylactic strategies, including surgical (mesh reinforcement) and non-surgical (abdominal wall strengthening exercises, AWSE) interventions, have been proposed, but their comparative effectiveness is unclear. This network meta-analysis primarily assessed PSH incidence.

**Methods:**

Following PRISMA guidelines, we conducted a systematic review and network meta-analysis. Searches in PubMed, Embase and Web of Science identified randomised controlled trials (RCTs) and observational studies comparing prophylactic PSH prevention strategies. Data on PSH incidence were extracted. Network meta-analysis estimated odds ratios (ORs) and 95% confidence intervals (CIs). Effectiveness was determined by PSH incidence reduction, comparing all prophylactic interventions against a transrectal colostomy control group. Interventions were ranked using surface under the cumulative ranking curve probabilities.

**Results:**

The analysis included 73 studies (30 RCTs, 44 observational; 7473 patients). Funnel mesh was the most effective intervention (OR 0.09, 95% CI 0.05–0.17), followed by Stapled Mesh stomA Reinforcement Technique (SMART) (OR 0.16, 95% CI 0.05–0.48) and AWSE (OR 0.18, 95% CI 0.08–0.39). Subgroup analyses confirmed consistency in findings across study designs but highlighted variability in ileal conduits due to limited data. Heterogeneity was moderate (*τ*^2^ = 0.21, *I*^2^ = 36.1%).

**Conclusions:**

Funnel mesh could be the most effective measure for high-risk patients, while extraperitoneal colostomy (ES) and AWSE may be a practical and scalable alternative. Further high-quality RCTs are needed to validate these findings and refine clinical guidelines for PSH prevention.

**Supplementary Information:**

The online version contains supplementary material available at 10.1007/s10151-025-03211-6.

## Introduction

Parastomal hernia (PSH) is a common complication in ostomized patients, with incidence rates ranging from 10% to 100%, depending on the length of follow-up and differences in study design [1, 2]. The progressive enlargement of the stoma trephine over time has led some researchers to suggest that PSH may be inevitable [3].

PSH is associated with significant morbidity, including difficulties securing ostomy appliances, leading to dermatitis, pain and challenges with stoma evacuation. Additionally, complications similar to those observed in incisional hernias, such as strangulated hernia, may occur. Approximately 30% of patients with PSH require surgical repair, further increasing the physical and psychological burden. These factors collectively contribute to a significant reduction in quality of life, highlighting the urgent need for effective prophylactic strategies.

Several factors increase the risk of PSH, including female sex, chronic obstructive pulmonary disease (COPD), length of temporary stoma, abdominal wall atrophy, large stoma sizes (> 30 mm) and peristomal infections [4–6]. To mitigate these risks, a variety of prophylactic measures have been proposed, including surgical techniques such as stoma suturing, modifying stoma site [7], extraperitoneal approach [8] and mesh implantation during initial surgery [9–12]. Non-surgical approaches, such as strengthening the abdominal wall musculature [13], have also been suggested but remain underexplored.

Given the variability in prophylactic strategies and the challenges of conducting large-scale randomised controlled trials (RCTs) for each intervention, a network meta-analysis provides a robust and comprehensive method to evaluate the relative effectiveness of available options. This approach integrates direct and indirect evidence from a connected network of comparisons, enhancing statistical power and facilitating the ranking of interventions based on efficacy [14].

This study aims to identify the most effective prophylactic measures for reducing the risk of PSH in ostomized patients. By synthesizing direct and indirect evidence, it seeks to provide robust, evidence-based recommendations to guide clinical decision-making and improve patient outcomes.

## Patients and methods

The protocol of this study was registered in the PROSPERO registry (CRD420245331620) and the guidelines of PRISMA, as amended for network meta-analysis, were followed.

### Search strategy

A comprehensive search was performed in the electronic bibliographic databases PubMed, Embase and Scopus. The search criteria for articles for this meta-analysis were the MeSH descriptors together with the title of the article. The terms used were “parastomal hernia”, “stoma”, “ostomized”, “colostomy”, “ileostomy”, “urostomy”, “incisional hernia”, “extraperitoneal”, “intraperitoneal”, “transperitoneal”, “retromuscular”, “transrectal”, “lateral” and “mesh”. Boolean combinations were combined with these terms:

[parastomal hernia] & ([extraperitoneal] OR [intraperitoneal] OR [transperitoneal] OR [retromuscular] OR [transrectal] OR [intraperitoneal] OR [transperitoneal] OR [transrectal] or [lateral] or [mesh]).

([incisional hernia] AND ([stoma] OR [ostomized] OR [colostomy] OR [ileostomy] OR [urostomy])) & ([extraperitoneal] OR [intraperitoneal] OR [transperitoneal] OR [retromuscular] OR [transrectal] OR [intraperitoneal] OR [transperitoneal] OR [transrectal] or [lateral] or [mesh]).

The search criteria did not include any language or time restrictions.

### Inclusion criteria

Three independent authors (VALC, JMA and DMV) systematically searched the three pre-specified bibliographic databases. Studies selection was conducted in a rigorous three-phase process based on predefined PICO eligibility criteria [15]. Initially, titles were independently screened to identify potentially relevant studies. Subsequently, abstracts of the selected titles underwent a thorough, independent review. Finally, full texts of all eligible abstracts were retrieved and meticulously assessed for final inclusion.

The PICO eligibility criteria for inclusion were as follows: the study population comprised patients aged over 18 years requiring either temporary or permanent stomas; interventions encompassed prophylactic measures for PSH prevention, including abdominal wall support garments, abdominal wall strengthening exercises (AWSE), stoma placement techniques, stoma site selection and mesh use; studies reported a minimum 6-month follow-up; and the primary outcome was the incidence of PSH.

Discrepancies arising from the independent review process were resolved by consensus among the three authors, or, if necessary, by arbitration with a fourth author (VPM).

The characteristics of each included study were systematically extracted and recorded on a specially designed meta-analysis form (Microsoft Excel 2016). Study variables included author, publication year, type of prophylaxis, PSH incidence, sample size, pathology type (gastrointestinal/urinary), minimum follow-up time, mesh location, mesh type and study design (RCT or NRCT). Meshes were further categorized by implant location and specific type (flat or FM).

The diagnosis of PSH incidence in the included studies was based on clinical examination, radiological findings via computed tomography (CT) scan, or a combination of both.

### Definition of surgical techniques

The surgical techniques are defined in Supplementary Material 1 (Table S1).

### Risk of bias and quality assessment

All papers were independently graded using the ROBINS I tool by two authors (JMA and VPM). A third author (DMV) confirmed the final decision through discussion.

The GRADE methodology [16–19] was used to assess the overall quality of evidence. GRADE tables were developed separately for clinical trials and observational studies to ensure a more accurate assessment of the quality of evidence, given the inherent methodological differences between these study designs. This approach was necessary to take into account the different levels of bias, precision and applicability that are associated with randomised and non-randomised studies.

### Assessment of risk of publication bias

To detect and manage publication bias, funnel plots and statistical tests such as Egger's test were used, with a* p* value < 0.10 considered indicative of potential bias.

### Statistical analysis

A frequentist network meta-analysis evaluated prophylactic measures for PSH prevention. Using a random-effects model, this approach enabled direct and indirect comparisons. Network density and connectivity were visually assessed. Consistency was evaluated globally via Wald’s test and for local inconsistencies using Bucher's method and netsplit plots.

Heterogeneity was quantified using the *I*^2^ statistic to estimate the proportion of total variability attributable to heterogeneity, alongside a heterogeneity coefficient calculated for each pairwise comparison. Forest plots were generated to visually represent the effect sizes and heterogeneity estimates for individual studies as well as the pooled results, providing a clear summary of the evidence.

Rankograms and P-score were developed to depict the cumulative probability of each intervention’s efficacy, offering a detailed ranking of the prophylactic measures for PSH prevention.

Subgroup analyses were performed on the basis of study design (RCTs vs observational studies), on specific interventions, such as ileal conduits or digestive stomas, diagnostic method of PSH and time of follow-up to ensure robust interpretation of the data.

Direct evidence diagrams, generated by the network meta-analysis, and netsplit plots were included to illustrate the strength and coherence of comparisons within the network.

All statistical analyses were performed using RStudio software (version 4.3.3) with the dmetar, meta and netmeta libraries. Risk of bias graphics were made with robvis tool [20]. Statistical significance was defined as *p* ≤ 0.05 in two-tailed tests.

## Results

### Description of included studies

**Fig. 1:**
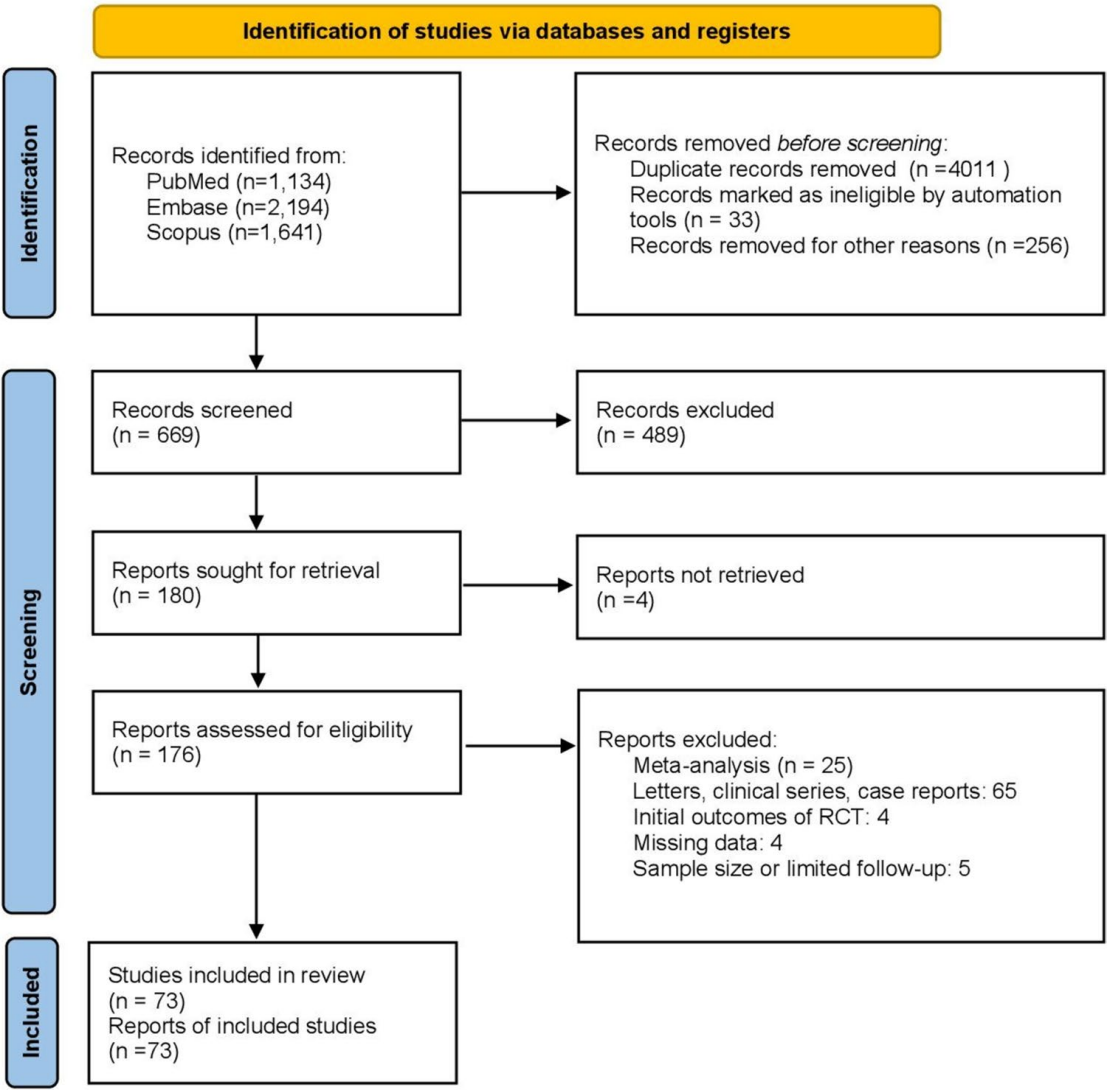
Flow diagram of the selected studies included in the network meta-analysis

The network meta-analysis included a total of 73 studies (Fig. 1), comprising 29 RCTs and 44 observational studies. These studies included 7473 patients, with an overall PSH incidence of 24.85% (*n* = 1857). Eight prophylactic strategies were analysed: funnel mesh (FM) [21–27], SMART [11, 28, 29], AWSE [13, 30, 31], ES (extraperitoneal colostomy) [16–19, 32–47], SM (sublay mesh) [48–65], intraperitoneal mesh (IPM) [2, 29, 66–70], transrectal stoma (control) and lateral stoma (LS) [3, 35, 46, 71–82]. Of the 73 head-to-head comparisons that were included, the most common were control vs ES (*n* = 24 studies, 1293 patients) and control vs SM (*n* = 17 studies, 856 patients).

The pooled incidence of PSH, based on the included studies, demonstrated varied rates across different prophylactic strategies. The control group exhibited an incidence of 30.8% (1257/4078). Among the interventions, ES recorded the lowest pooled incidence at 4.7% (61/1293). The incidence rates for other techniques were as follows: AWSE 10.9% (27/248); FM 9.1% (22/241); SMART 2.2% (9/74); LS 28.2% (153/542); IPM 37.6% (53/141); and SM 32.1% (275/856).

A significant proportion of the included studies were from Europe and Asia and spanned several geographical regions. Follow-up periods varied widely, from 6 months to over 5 years, which may have had an impact on the reported incidence of PSH. Methodological quality varied between studies. While most RCTs followed robust protocols, observational studies showed greater heterogeneity in design and reporting. In particular, variability in the use of the SM was observed, with some studies using different synthetic materials [49–51, 53–55, 57, 58, 60, 62–65, 83] and others using resorbable meshes [48, 52, 56, 59, 61], which may have influenced the results.

Table 1 summarises the main characteristics of the studies used in this meta-analysis.

**Table 1:** Summary of studies included in network meta-analysis

Study	Type of study	Prophylactic measure	Composition of mesh	Type of stoma	Pathology	Surgical approach	PH diagnosis	Outcomes		Follow-up
								PH	Total	
Extraperitoneal stoma										
Harshaw-1974	Retrospective	Extraperitoneal stoma	–	Colostomy	Malign	Open	Clinical	0	17	?
		Control						9	82	
Marks-1975	Retrospective	Extraperitoneal stoma	–	Colostomy	Malign	Open	Clinical	1	37	60
		Control						22	190	60
Whittaker-1976	Retrospective	Extraperitoneal stoma	–	Colostomy	Malign		Clinical	8	89	24
		Control						28	162	24
von Smitten-1986	Retrospective	Extraperitoneal stoma	–	Colostomy	Malign	Open	Clinical	5	12	54 (12–96)
		Control						21	42	
Hwang-1990	Retrospective	Extraperitoneal stoma	–	Colostomy	Mixed	Open	Clinical	1	41	20
		Control						2	138	
Londono-Schimmer-1994	Retrospective	Extraperitoneal stoma	–	Colostomy	Malign	Open	Clinical	1	28	66 (2–250)
		Control						24	170	
		Lateral stoma						7	31	
Ding-2007	Retrospective	Extraperitoneal stoma	–	Colostomy	Malign	Laparoscopic	Clinical	0	26	12
		Control						3	37	
Qu-2008	Retrospective	Extraperitoneal stoma	–	Colostomy	Malign	Laparoscopic	Clinical	0	30	22
		Control		Colostomy				1	30	
Yang-2010	Retrospective	Extraperitoneal stoma	–	Colostomy	Malign	Laparoscopic	Clinical	0	75	33
		Control						4	37	
Dong-2012	RCT	Extraperitoneal stoma	–	Colostomy	Malign	Open	Clinical + CT	0	66	60
		Control						3	62	
Hamada-2008	Retrospective	Extraperitoneal stoma	–	Colostomy	Malign	Laparoscopic	CT	1	22	24
		Control						5	15	14
Leroy-2012	Retrospective	Extraperitoneal stoma	–	Colostomy	Malign	Laparoscopic	Clinical	0	12	?
		Control						4	10	
Gong-Yiang-2013	Retrospective	Extraperitoneal stoma	–	Colostomy	Malign	Laparoscopic	CT	0	21	12–36
		Control						5	21	
Xue-Feng-2013	Retrospective	Extraperitoneal stoma	–	Colostomy	Malign	Laparoscopic	Clinical + CT	0	28	24
		Control						3	32	
Funahashi-2014	Retrospective	Extraperitoneal stoma	–	Colostomy	Malign	Both	Clinical + CT	6	46	31 (0.5–91)
		Control						16	34	
Jin Heiying-2014	RCT	Extraperitoneal stoma	–	Colostomy	Malign	Laparoscopic	Clinical	0	18	17 (12–24)
		Control						2	18	
Ye-2014	RCT	Extraperitoneal stoma	–	Colostomy	Malign	Laparoscopic	Clinical	0	41	12
		Control						3	40	
Zhou Haitao-2016	RCT	Extraperitoneal stoma	–	Colostomy	Malign	Laparoscopic	CT	0	35	12
		Control						4	34	
Wu Jindong-2017	RCT	Extraperitoneal stoma	–	Colostomy	Malign	Laparoscopic	CT	0	53	12
		Control						3	53	
Ota-2022	Retrospective	Extraperitoneal stoma	–	Colostomy	Malign	Laparoscopic	Clinical + CT	2	105	24
		Control						38	222	
Xiao-2023	Retrospective	Extraperitoneal stoma	–	Colostomy	Malign			14	103	33
		Control					Clinical + CT	76	202	
Tanaka-2024	Retrospective	Extraperitoneal stoma	–	Ileal conduit	Malign	Laparoscopic	CT	2	57	22
		Control						9	46	
Zhang-2024	Retrospective	Extraperitoneal stoma	–	Colostomy	Malign	Laparoscopic	CT	0	37	36
		Control						8	37	
Lateral stoma										
Eldrup-1982	Retrospective	Lateral stoma	–	Colostomy	Mixed	Open	Clinical	12	63	6
		Control						1	77	
von Smitten-1986	Retrospective	Lateral stoma		Colostomy	Malignant	Open	Clinical	15	26	12–96
		Control						10	25	
Sjödahl-1988	Retrospective	Lateral stoma	–	Colostomy, ileostomy	Mixed	Open	Clinical	6	23	84 (12–432)
		Control						3	107	
Williams-1990	Retrospective	Lateral stoma	–	Ileostomy	Benign	Open	Clinical + CT	6	16	78
		Control						4	12	
Leong-1994	Retrospective	Lateral stoma	–	Ileostomy	Mixed	Open	Clinical	2	42	110.4 (3.6–240)
		Control						14	103	
OrtIz-1994	Retrospective	Lateral stoma	–	Colostomy	Malignant	Open	Clinical + CT	15	29	60
		Control						11	25	
Cingi-2006	Retrospective	Lateral stoma	–	Colostomy, ileostomy	Mixed	Open	CT	5	6	15 (2–63)
		Control						12	14	
Pilgrim-2010	Retrospective	Lateral stoma	–	Colostomy, ileal conduit, ileostomy	Mixed	Open	Clinical	1	10	14.1
		Control						29	80	
Hardt-2015	RCT	Lateral stoma	–	Ileostomy	Malignant	Open, laparoscopic	Surgery	5	27	127 (57–306)
		Control						4	29	112 (15–642)
Ho-2018	Retrospective	Lateral stoma	–	Colostomy	Malignant	Laparoscopic	CT	11	12	24 (0–90)
		Control						78	91	
Li-2022	Retrospective	Extraperitoneal stoma	–	Ileal conduit	Malignant	Open	CT	13	214	24 (6–180)
		Lateral stoma					CT	30	161	
Soomro-2022	Retrospective	Lateral stoma	–	Colostomy	Malignant	Laparoscopic, open	CT	22	36	?
		Control					CT	16	47	
Zhou-2024	RCT	Extraperitoneal stoma	–	Ileal conduit	Malign	Laparoscopic	CT	6	52	24
		Lateral stoma						15	52	
Abdominal training exercises										
Thompson-2007	Retrospective	Exercises	–	All	Mixed	Open	CT	16	114	12
		Control						24	87	12
Yang-2015	RCT	Exercises	–	Colostomy	Malignant	Laparoscopic	CT	7	105	6
		Control						27	105	6
Lopez-Callejon-2025	Retrospective	Exercises	–	All	Mixed	Laparoscopic, open	Clinical + CT	4	29	12
		Control						33	36	
Funnel mesh										
Jánó-2014	Retrospective	Funnel mesh	Polypropylene, PG/polypropylene	Colostomy	Malignant	Laparoscopic	CT	0	13	12
		Control	–				CT	4	13	12
Jánó-2016	RCT	Funnel mesh	Polypropylene	Colostomy	Malignant	Laparoscopic, open	Clinical + CT	3	38	19
		Control	–					18	46	22
López-Borao-2019	Retrospective	Funnel mesh	Polyvinylidene fluoride + polypropylene	Colostomy	Malignant	Laparoscopic	Clinical + CT	6	46	33.6
		Control	–					46	64	33.6
Ammann-2021	Retrospective	Funnel mesh	Polyvinylidene fluoride + polypropylene	Colostomy	Malignant	Laparoscopic, open	CT	2	22	12
		Control	–				CT	23	54	12
Arrayas-2023	Retrospective	Funnel mesh	Polyvinylidene fluoride + polypropylene	Colostomy	Malignant	Laparoscopic	CT	2	30	18–24
		Control	–				CT	19	30	
Badia-Closa-2024	Retrospective	Funnel mesh	Polyvinylidene fluoride + polypropylene	Colostomy	Malignant	Laparoscopic	Clinical + CT	3	34	22.06
		Control	–					26	38	63.18
Mäkäräinen-2024	RCT	Funnel mesh	Polyvinylidene fluoride + polypropylene	Colostomy	Malignant	Laparoscopic, robotic	CT	6	58	12
		Control	–					22	59	12
SMART/STORMM technique										
Williams-2015	Retrospective	SMART	Porcine-derived acellular dermal sheet	Colostomy	Malignant	Laparoscopic, open	Clinical + CT	4	21	
		Control	–					8	11	
Canda-2018	Retrospective	SMART	Polypropylene	Colostomy	Malignant	Laparoscopic, open	CT	4	29	24
		Control	–				CT	15	38	24
Sublay mesh										
Hammond-2008	RCT	Sublay mesh	Porcine-derived acellular dermal sheet	Loop ileostomy	Mixed	Open	Clinical	0	10	12
		Control	–					3	10	
Serra-Aracil-2009	RCT	Sublay mesh	Polyglecaprone–polypropylene	Colostomy	Malignant	Open	CT	9	20	29
		Control	–				CT	15	24	
Jänes-2009	RCT	Sublay mesh	PG–polypropylene	Colostomy	Mixed	Open	Clinical	2	27	60
		Control						17	27	60
Ventham, 2012	Retrospective	Sublay mesh	Polypropylene	Colostomy	Malignant	Open	CT	10	17	12
		Control	–					14	24	12
Fleshman-2014	RCT	Sublay mesh	Non-cross-linked porcine acellular dermal matrix	Colostomy, ileostomy	Malignant	Open, laparoscopic	Clinical + CT	5	49	24
		Control	–					7	53	
Tarcoveanu-2014	RCT	Sublay mesh	Polypropylene	Colostomy	Malignant	Open	Clinical	0	20	20
		Control	–					6	22	22
Lambrecht-2015	RCT	Sublay mesh	Polypropylene	Colostomy	Malignant	Open	Clinical + CT	2	32	36
		Control	–					12	26	48
Nikberg-2015	Retrospective	Sublay mesh	Polygalactin/polypropylene, polyester–polylactic acid	Colostomy	Malignant	Open	Clinical + CT	34	66	31 (12–202)
		Control	–					49	115	
Tenzel-2018	Retrospective	Sublay mesh	Phasix®	Ileal conduit	Malignant	Robotic	Clinical + CT	0	18	21
		Control						1	20	21
Odensten-2019	RCT	Sublay mesh	Polypropylene	Colostomy	Malignant	Open	Clinical + CT	33	104	12
		Control						36	107	
Liedberg-2020	RCT	Sublay mesh	Polypropylene	Ileal conduit	Malignant	Open	Clinical + CT	12	118	36 (24–60)
		Control	–					26	123	36 (24–48)
Correa-Marinez-2021	RCT	Sublay mesh	Polypropylene–polyglecaprone	Colostomy	Malignant	Laparoscopic, open	CT	23	58	12
		Control	–				CT	32	63	
Prudhomme-2021	RCT	Sublay mesh	Polypropylene	Colostomy	Mixed	Laparoscopic, open	Clinical + CT	30	70	24
		Control	–					28	65	
Adasonla-2022	Retrospective	Sublay mesh	Biological	Colostomy	Mixed	Unspecified	CT	35	55	40 (22–59)
		Control	–					49	71	
Frigault-2022	Retrospective	Sublay mesh	Polypropylene	Colostomy, ileostomy	Mixed	Laparoscopic, Open	CT	32	79	19 (10.8–30.6)
		Control	–					47	106	
Pizza-2022	RCT	Sublay mesh	Polyglycolide–trimethylene carbonate copolymer	Colostomy	Mixed	Open	CT	9	55	12
		Control	–					24	55	
Ringblom-2022	RCT	Sublay mesh	Polypropylene	Colostomy	Mixed	Laparoscopic, open	Clinical + CT	42	99	19.2 (10.9–32.9)
		Control	–					45	101	
Gao-2022	Retrospective	Sublay mesh	Polypropylene	Colostomy	Malignant	Laparoscopic	CT	10	24	20
		Control	–					23	32	
Brandsma-2023	RCT	Sublay mesh	Polypropylene	Colostomy	Malignant	Laparoscopic, open	Clinical + CT	20	51	12
		Control	–					29	54	
Intraperitoneal mesh										
Cui-2009	RCT	Intraperitoneal mesh	Expanded polytetrafluoroethylene	Colostomy	Malignant	Open	Clinical + CT	0	30	36 m
		Control						8	30	
López-Cano-2012	RCT	Intraperitoneal mesh	Polydioxanone coated + polypropylene	Colostomy	Malignant	Laparoscopic	Clinical + CT	9	18	10.4
		Control						15	16	
Vierimaa-2015	RCT	Intraperitoneal mesh	Polypropylene–polyvinylidene fluoride	Colostomy	Malignant	Laparoscopic	Clinical + CT	18	35	12
		Control	–					17	35	
López-Cano-2016	RCT	Intraperitoneal mesh	Polypropylene–polyglecaprone	Colostomy	Malignant	Laparoscopic	CT	10	18	19
		Control						19	26	17
Mäkäräinen-Uhlbäch-2020	RCT	Intraperitoneal mesh	Polypropylene	Colostomy	Malignant	Laparoscopic	Clinical + CT	9	19	69.6 ± 12.7
		Control	–					7	12	67.3 ± 9.2
Fox-2021	Retrospective	Intraperitoneal mesh	Macroporous polypropylene	Colostomy	Mixed	Open, laparoscopic, robotic	Clinical + CT	5	28	16
		SMART						1	24	16
Djaladat-2024	RCT	Intraperitoneal mesh	FlexHD structural biological mesh	Ileal conduit	Malignant	Open, robotic	Clinical + CT	18	72	24
		Control	–					19	74	24

*CT* computed tomography,* RCT* randomised clinical trial,* SMART* Stapled Mesh stomA Reinforcement Technique

### Quality and risk of bias

The assessment of the quality of the studies and their risk of potential errors was performed on a case-by-case basis according to the type of study: RCT (Supplementary Material 1, Fig. S1) or NRCT (Supplementary Material 1, Fig. S2).

### Network

**Fig. 2:**
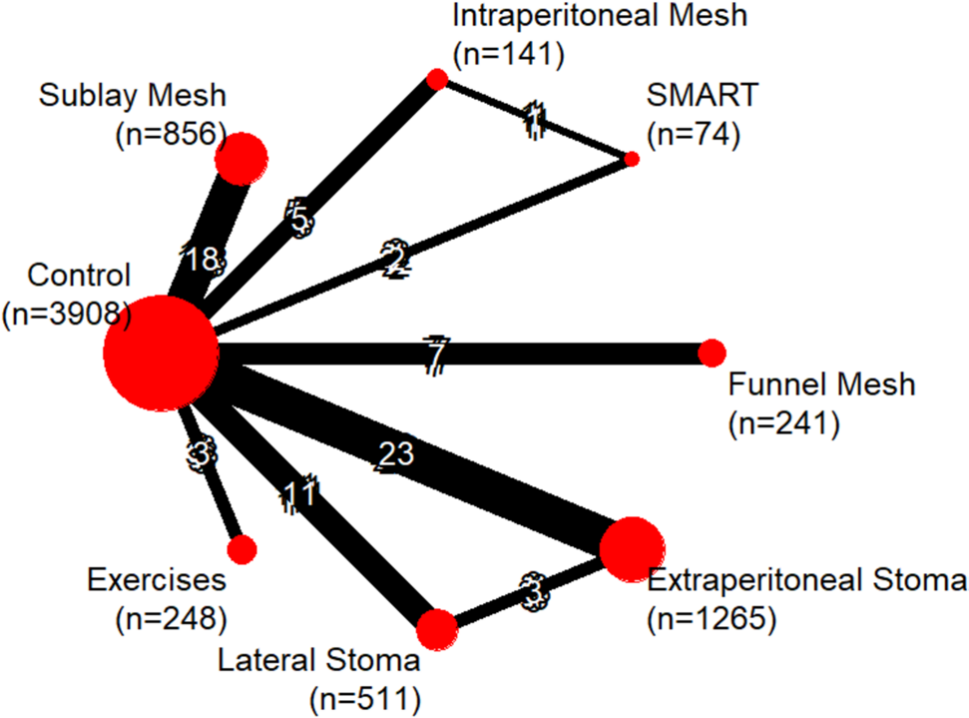
Design of the meta-analysis network of studies

A total of 73 comparisons were included (Fig. 2), with extraperitoneal route of stoma versus SM being the most commonly evaluated.

In the case of direct comparisons, the largest number of included studies corresponded to the control vs ES comparison (*n* = 24), which included a total of 1283 patients, and the control vs SM comparison (*n* = 18), which included 842 patients.

### Network outcomes

The network meta-analysis revealed moderate heterogeneity across the included studies, with a *τ*^2^ of 0.21 and *I*^2^ of 36.1% (95% CI 13.6–52.8%). Tests of heterogeneity within designs (*Q* = 101.76, *p* = 0.0024) showed statistically significant variability, while the test for inconsistency between designs (*Q* = 3.18, *p* = 0.5288) revealed no significant discrepancies between direct and indirect comparisons. These results suggested that the network was consistent and the estimates were reliable for comparing prophylactic techniques to control group.

### Efficacy of prophylactic interventions

**Fig. 3:**
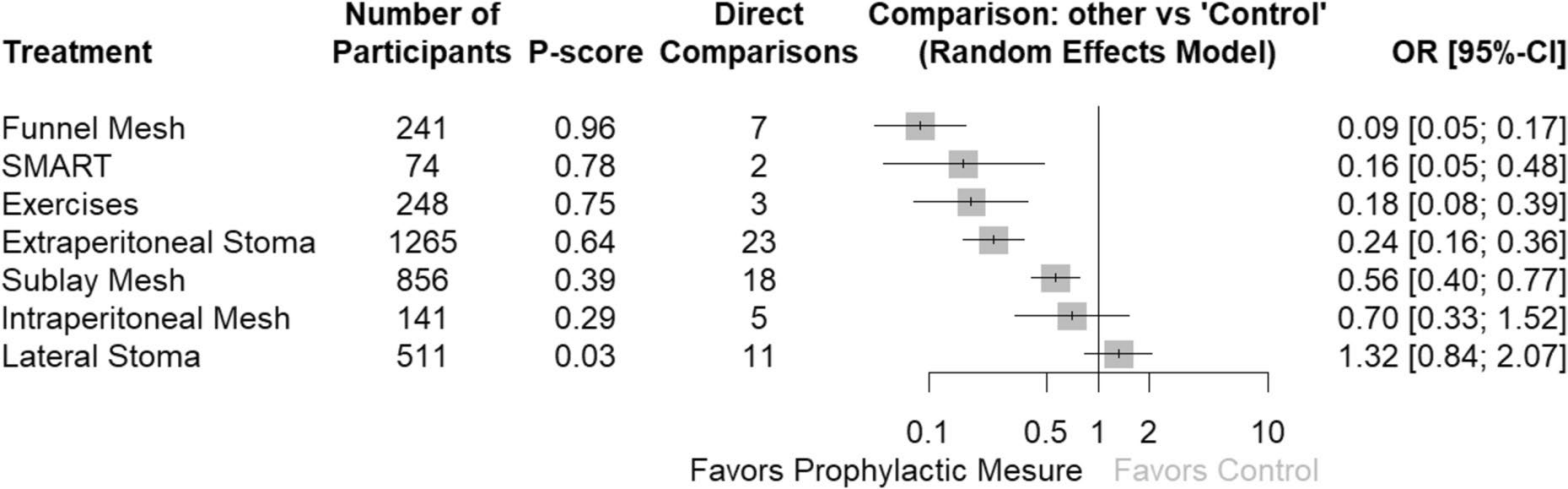
Forest plot of the comparisons between prophylactic measures to reduce the risk of parastomal hernia versus control group (transrectal stomas)

The forest plot comparing prophylactic measures to reduce the risk of PSH versus control (Fig. 3) showed that the order by risk and P-score was FM, SMART, AWSE, ES and SM. No significant difference was observed between IPM and LM in their ability to reduce the risk of PSH compared to the control group.

**Fig. 4:**
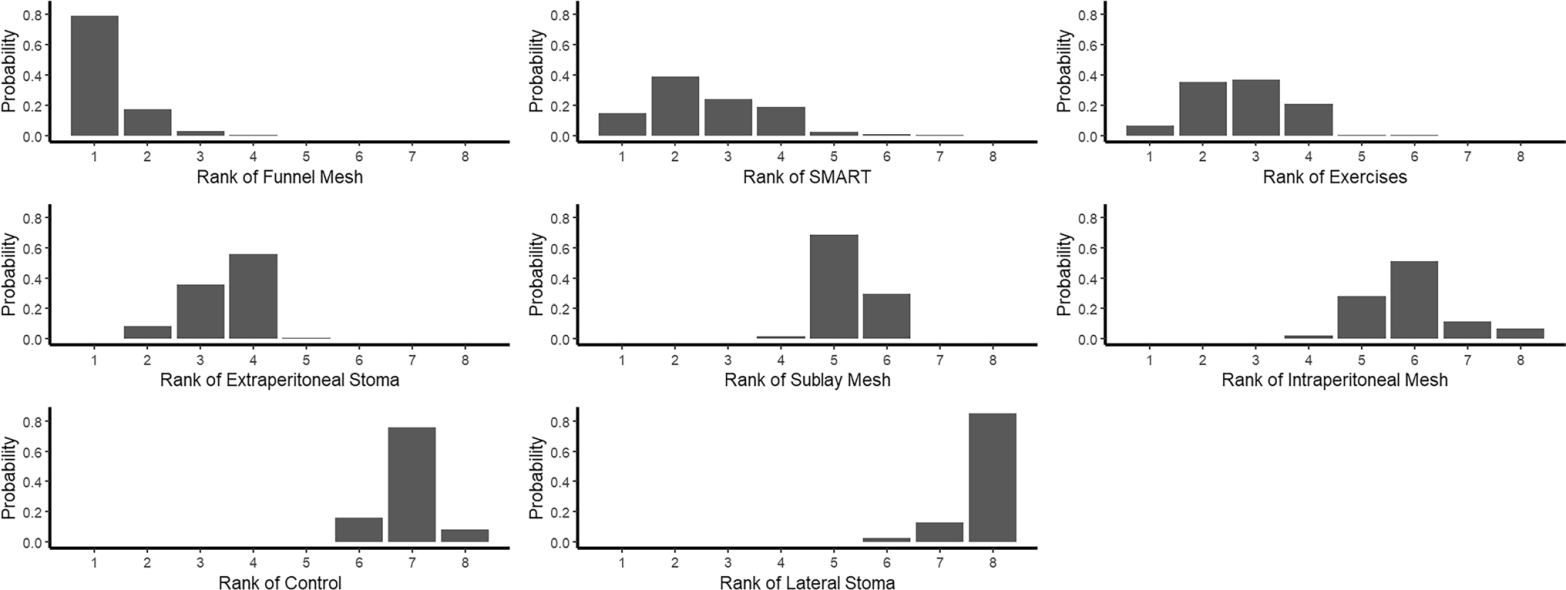
Rankogram of all prophylactic measures to decrease the risk of parastomal hernia in network meta-analysis

The cumulative rank plots and rankograms (Fig. 4) consistently ranked FM, SMART technique, AWSE, ES, SM, IPM, control and LM as the most to least effective prophylactic measures for reducing PSH risk. Subgroup analysis stratified by study design (RCTs and observational studies) confirmed the consistency of the top four interventions (FM, SMART, AWSE and ES) across both subgroups (Supplementary Material 2, Figs. S1–S2).

The greatest reduction in PSH risk was observed with FM (OR 0.09, 95% CI 0.05–0.17), SMART technique (OR 0.16, 95% CI 0.05–0.48), AWSE (OR 0.18, 95% CI 0.08–0.39), ES (OR 0.24, 95% CI 0.16–0.36) and SM (OR 0.56, 95% CI 0.40–0.77). Other prophylactic measures did not demonstrate a significant reduction in PSH risk (Table 2). A comparison of the effective interventions (Table 3) revealed that only FM versus AWSE (OR 2.0, 95% CI 0.74–5.42) showed no significant differences.

**Table 2:** Comparison of the outcomes of the direct and indirect evidence from all the studies in the network meta-analysis (NMA)

Arm 1	Arm 2	*k*	*n*	*I*^2^	Direct estimate	Indirect estimate	NMA	Incoherence *p* value
Exercises	Control	3	476	86.4%	0.18 [0.08; 0.39]		0.18 [0.08; 0.39]	
Extraperitoneal stoma	Control	23	2713	0.0%	0.21 [0.14; 0.33]	0.46 [0.16; 1.29]	0.24 [0.16; 0.36]	0.18
Funnel mesh	Control	7	545	0.0%	0.09 [0.05; 0.17]		0.09 [0.05; 0.17]	
Intraperitoneal mesh	Control	5	238	40.5%	0.69 [0.31; 1.55]	0.83 [0.06; 12.17]	0.70 [0.33; 1.52]	0.90
Lateral stoma	Control	11	971	37.4%	1.55 [0.93; 2.57]	0.72 [0.27; 1.94]	1.32 [0.84; 2.07]	0.18
SMART	Control	2	99	0.0%	0.17 [0.05; 0.55]	0.14 [0.01; 1.75]	0.16 [0.05; 0.48]	0.90
Sublay mesh	Control	18	1841	56.5%	0.56 [0.40; 0.77]		0.56 [0.40; 0.77]	
Exercises	Extraperitoneal stoma	0				0.74 [0.31; 1.77]	0.74 [0.31; 1.77]	
Exercises	Funnel mesh	0				2.00 [0.74; 5.42]	2.00 [0.74; 5.42]	
Exercises	Intraperitoneal mesh	0				0.25 [0.08; 0.76]	0.25 [0.08; 0.76]	
Exercises	Lateral stoma	0				0.14 [0.06; 0.33]	0.14 [0.06; 0.33]	
Exercises	SMART	0				1.11 [0.29; 4.23]	1.11 [0.29; 4.23]	
Exercises	Sublay mesh	0				0.32 [0.14; 0.74]	0.32 [0.14; 0.74]	
Extraperitoneal stoma	Funnel mesh	0				2.71 [1.28; 5.74]	2.71 [1.28; 5.74]	
Extraperitoneal stoma	Intraperitoneal mesh	0				0.34 [0.14; 0.82]	0.34 [0.14; 0.82]	
Extraperitoneal stoma	Lateral stoma	3	538	0.0%	0.27 [0.12; 0.61]	0.14 [0.07; 0.28]	0.18 [0.11; 0.31]	0.24
Extraperitoneal stoma	SMART	0				1.51 [0.47; 4.83]	1.51 [0.47; 4.83]	
Extraperitoneal stoma	Sublay mesh	0				0.43 [0.26; 0.73]	0.43 [0.26; 0.73]	
Funnel mesh	Intraperitoneal mesh	0				0.13 [0.05; 0.34]	0.13 [0.05; 0.34]	
Funnel mesh	Lateral stoma	0				0.07 [0.03; 0.15]	0.07 [0.03; 0.15]	
Funnel mesh	SMART	0				0.56 [0.16; 1.95]	0.56 [0.16; 1.95]	
Funnel mesh	Sublay mesh	0				0.16 [0.08; 0.32]	0.16 [0.08; 0.32]	
Intraperitoneal mesh	Lateral stoma	0				0.53 [0.22; 1.31]	0.53 [0.22; 1.31]	
Intraperitoneal mesh	SMART	1	52		5.00 [0.45; 55.28]	4.19 [0.98; 17.87]	4.39 [1.27; 15.21]	0.90
Intraperitoneal mesh	Sublay mesh	0				1.26 [0.54; 2.91]	1.26 [0.54; 2.91]	
Lateral stoma	SMART	0				8.23 [2.53; 26.76]	8.23 [2.53; 26.76]	
Lateral stoma	Sublay mesh	0				2.36 [1.35; 4.12]	2.36 [1.35; 4.12]	
SMART	Sublay mesh	0				0.29 [0.09; 0.89]	0.29 [0.09; 0.89]

**Table 3:** Pairwise comparison of prophylactic measures to decrease the risk of parastomal hernia from network meta-analysis

Control							
5.61 (2.59; 12.16)	Exercises						
4.14 (2.75; 6.23)	0.74 (0.31; 1.77)	Extraperitoneal stoma					
11.24 (6.00; 21.04)	2.00 (0.74; 5.42)	2.71 (1.28; 5.74)	Funnel mesh				
1.42 (0.66; 3.08)	0.25 (0.08; 0.76)	0.34 (0.14; 0.82)	0.13 (0.05; 0.34)	Intraperitoneal mesh			
0.76 (0.48; 1.19)	0.14 (0.06; 0.33)	0.18 (0.11; 0.31)	0.07 (0.03; 0.15)	0.53 (0.22; 1.31)	Lateral stoma		
6.24 (2.10; 18.56)	1.11 (0.29; 4.23)	1.51 (0.47; 4.83)	0.56 (0.16; 1.95)	4.39 (1.27; 15.21)	8.23 (2.53; 26.76)	SMART	
1.79 (1.29; 2.48)	0.32 (0.14; 0.74)	0.43 (0.26; 0.73)	0.16 (0.08; 0.32)	1.26 (0.54; 2.91)	2.36 (1.35; 4.12)	0.29 (0.09; 0.89)	Sublay mesh

The analysis by study design confirmed that FM, AWSE and ES remained among the most effective measures across both RCTs and observational studies. However, the SMART technique, which showed strong efficacy in the overall analysis, was not evaluated in RCTs because of a lack of studies.

Subgroup analyses stratified by stoma types (colostomy vs ileal conduit) in the case of colostomies (Supplementary Material 2, Figs. S3–S4) showed results consistent with the global analysis, confirming FM as the most effective intervention (OR 0.09, 95% CI 0.05–0.17) and AWSE as a practical alternative (OR 0.18, 95% CI 0.08–0.39). In contrast, ileal conduits (Supplementary Material 2, Fig. S4) demonstrated variability in the choice of prophylactic measures due to the limited number of available studies. SM and AWSE showed moderate efficacy in this subgroup, but the lack of direct comparisons and the heterogeneity of studies highlight the need for further research.

A sensitivity analysis including only studies with a minimum follow-up of 24 months (*n* = 31 studies, 3757 patients) demonstrated consistent findings with the overall network, confirming the superior effectiveness of funnel mesh (OR 0.06, 95% CI 0.02–0.22) and extraperitoneal stoma (OR 0.26, 95% CI 0.16–0.41) in reducing PSH risk compared to the control. Sublay mesh also remained significantly protective (OR 0.47, 95% CI 0.29–0.79), while intraperitoneal mesh and lateral stoma did not show significant benefit. These results reinforce the robustness of the primary analysis across longer-term outcomes (Supplementary Material 2, Fig. S5).

To explore the potential influence of detection technique on outcomes, a sensitivity analysis was conducted including only studies that utilized CT for PSH diagnosis (*n* = 51; 5215 patients). The results confirmed the protective effect of the most effective interventions identified in the main analysis. Funnel mesh (OR 0.09, 95% CI 0.05–0.16), SMART (OR 0.16, 95% CI 0.06–0.45), AWSE (OR 0.20, 95% CI 0.10–0.38) and extraperitoneal stomas (OR 0.20, 95% CI 0.13–0.32) significantly reduced the risk of PSH compared to conventional transrectal stomas. Sublay mesh also showed a modest protective effect (OR 0.69, 95% CI 0.52–0.91), while intraperitoneal mesh and lateral stomas were not associated with statistically significant risk reduction. These findings suggest that the diagnostic method did not substantially alter the ranking or efficacy of the prophylactic strategies (Supplementary Material 2, Fig. S6).

### Consistency of evidence

The SIDE analysis (Table 2) assessed the coherence between direct and indirect evidence for key prophylactic interventions in PSH prevention. FM showed the most robust results, with consistent NMA and direct estimates (OR 0.09, 95% CI 0.05–0.17, *k* = 7). AWSE demonstrated a significant reduction in risk (OR 0.18, 95% CI 0.08–0.39, *k* = 3) and had 100% direct evidence, though high heterogeneity was noted (*I*^2^ = 86.4%). ES exhibited consistent results (OR 0.24, 95% CI 0.16–0.36, *k* = 23), with some variability between direct and indirect estimates (RoR = 0.47, *p* = 0.1799). SM showed moderate effectiveness (OR 0.56, 95% CI 0.40–0.77, *k* = 18) without contributions from indirect evidence. SMART showed a favourable OR (OR 0.16, 95% CI 0.05–0.48, *k* = 2) but was limited by a small evidence base. In contrast, IPM (OR 0.70, 95% CI 0.33–1.52, *k* = 5) and lateral stomas (OR 1.32, 95% CI 0.84–2.07, *k* = 11) were less effective, with higher variability.

A comparison of the effective interventions (Table 3) revealed that only FM versus AWSE (OR 2.0, 95% CI 0.74–5.42) showed no significant differences.

Overall, no significant incoherence was detected across the network, as all inconsistency tests yielded *p* > 0.05, reinforcing the robustness of the findings.

**Fig. 5:**
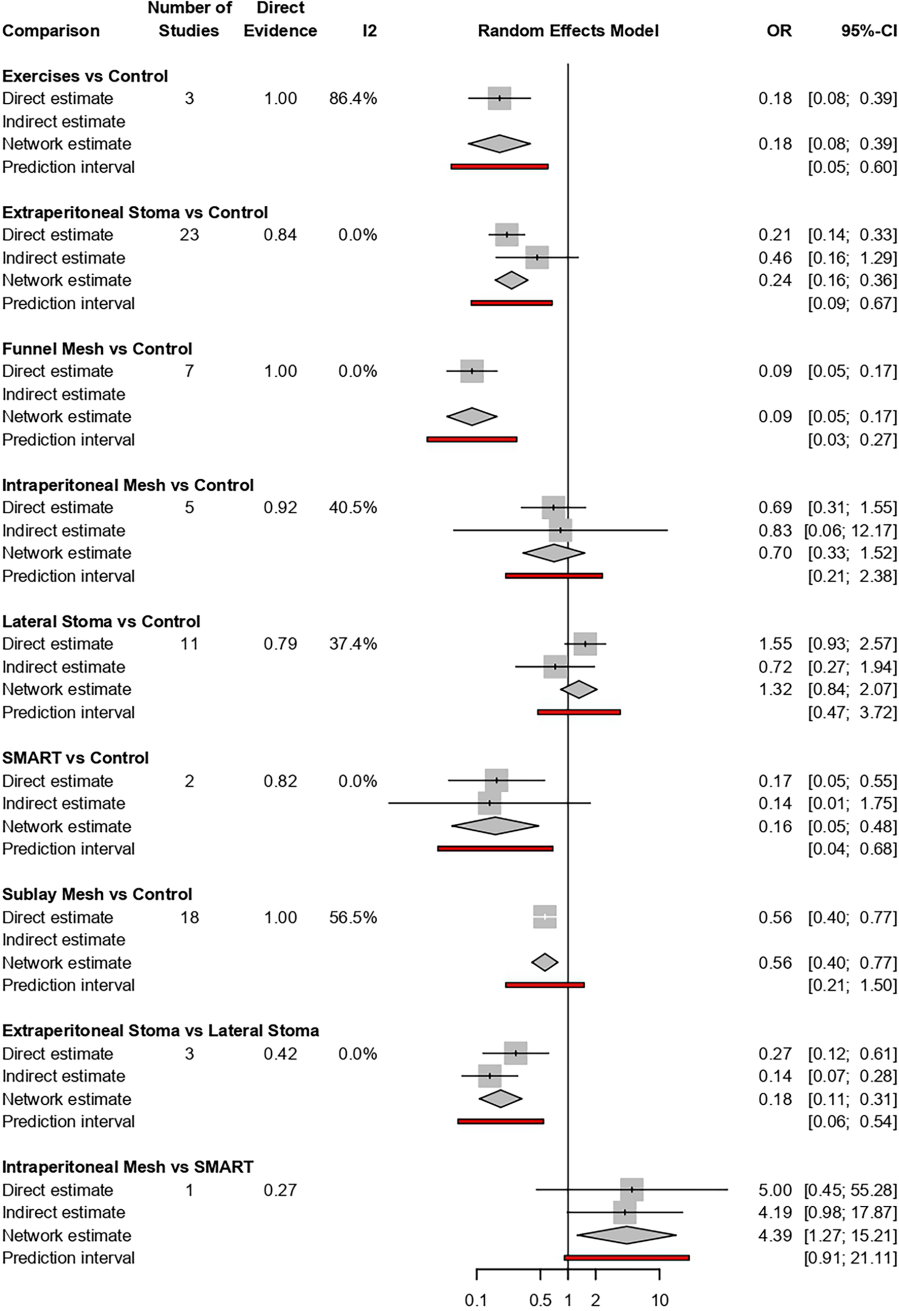
Netsplit analysis of the network meta-analysis comparing prophylactic measures for parastomal hernia prevention. The figure displays the consistency between direct and indirect evidence for each pairwise comparison. The* p* values represent tests for incoherence, with non-significant values (*p* > 0.05) indicating agreement between direct and indirect estimates. This analysis supports the overall robustness and reliability of the network

To assess the coherence between direct and indirect evidence, a netsplit analysis was performed (Fig. 5). The results demonstrated no significant incoherence (*p* > 0.05 for all comparisons), reinforcing the robustness of the network meta-analysis findings.

The design-specific decomposition of the *Q* statistic revealed significant heterogeneity in control versus AWSE (*Q* = 14.69, *p* = 0.0006) and control versus SM (*Q* = 39.08, *p* = 0.0017). In contrast, comparisons such as control versus SMART (*Q* = 0.90, *p* = 0.3439) and control versus FM (*Q* = 5.47, *p* = 0.4854) were consistent. The global test for consistency showed no significant inconsistency (*Q* = 1.84, *p* = 0.7644), confirming the robustness of the network and the agreement between direct and indirect comparisons.

### Bias analysis

**Fig. 6:**
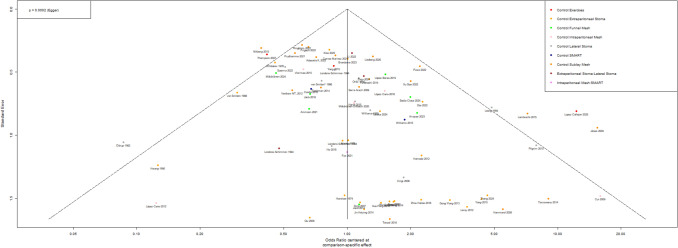
Funnel plot of publications included in network meta-analysis. Egger’s test confirmed publications bias

Egger's test indicated an asymmetry in the funnel plot of the comparisons in the network meta-analysis (*p* = 0.0002), which could indicate systematic bias in the available literature (Fig. 6).

## Grade evaluation of evidence quality

FM emerged as the most effective intervention for reducing PSH incidence. High-certainty evidence from RCTs demonstrated a significant risk reduction, from 381 to 95 per 1000 patients, with a median follow-up of 15.5 months. This finding was further supported by moderate-certainty evidence from retrospective studies, confirming its robust efficacy across different study designs.

AWSE were also highly effective, with high-certainty evidence from RCTs indicating a reduction in PSH risk from 257 to 68 per 1000 patients over a median follow-up of 6 months. These findings highlight the potential of AWSE as a practical and cost-effective alternative, particularly in settings where surgical resources are limited. Retrospective studies provided additional support for their protective role, albeit with moderate certainty due to variability in implementation and follow-up protocols.

ES demonstrated strong efficacy, with high-certainty evidence from RCTs showing a reduction in PSH risk from 72 to 15 per 1000 patients over 12 months. This finding underscores the potential of surgical innovation in PSH prevention, although its practical application may be limited by technical challenges, particularly in laparoscopic settings.

SM showed moderate efficacy, with high-certainty evidence from RCTs revealing a reduction in PSH risk from 387 to 221 per 1000 patients over a median follow-up of 24 months. However, the lower certainty of evidence from retrospective studies and variability in mesh materials suggest that its protective effect may depend on surgical context and implementation techniques.

In contrast, LS did not demonstrate significant efficacy in reducing PSH risk, with high-certainty evidence from RCTs and retrospective studies showing no meaningful differences compared to conventional stomas. The SMART-STORMM technique, while promising, was supported only by low-certainty evidence from retrospective studies. A reported reduction in PSH risk from 469 to 160 per 1000 patients suggests potential benefits, but the small number of studies, short follow-up durations and variability in surgical techniques limit the generalizability of these findings.

NMA grade profile and summary findings table are available in Supplementary Material 3 and 4.

## Discussion

The primary objective of this network meta-analysis was to provide an evidence-based ranking of prophylactic measures to reduce the risk of PSH. Inclusion of all available evidence made summarising the information very complex, as the studies used different definitions and diagnostic methods for PSH and had very different follow-up times. The analysis confirmed that transrectal stomas, routinely performed in clinical practice, are associated with a high PSH incidence, emphasizing the need to implement effective prophylactic strategies. Among the evaluated measures, FM emerged as the most effective intervention, supported by high-quality evidence from RCTs and moderate-quality evidence from observational studies.

Other mesh techniques, such as SMART and SM, demonstrated significant protective effects against PSH. SMART showed efficacy comparable to FM but was associated with higher resource utilization. SM, while protective, was less effective than FM and SMART. The IPM did not significantly reduce PSH risk compared to control groups, and its routine use cannot be recommended on the basis of the current evidence.

Despite their demonstrated efficacy and guidelines recommendations [84–87], prophylactic meshes remain underutilized, with only 7% of ostomy patients receiving them [88], primarily because of concerns about complications, increased operative time and inconsistent evidence supporting their use. Only 10% of colorectal surgeons routinely employ meshes [89], reflecting doubts about their benefits and concerns over infection, abdominal muscle wasting and the complexity of mesh implantation. This network meta-analysis addressed these gaps by individually analysing surgical approaches and stratifying mesh techniques, providing more nuanced insights into their efficacy.

Subgroup analyses confirmed the consistency and robustness of the protective role of the prophylactic measures identified in the global analysis. In patients undergoing ileal conduit creation, the analysis highlighted ES and SM as the two most effective interventions for reducing PSH risk. However, these findings should be interpreted with caution because of the limited number of studies available in this subgroup, resulting in a low level of recommendation for these measures. In contrast, in the colostomy subgroup efficacy of FM, SMART and AWSE was confirmed, consistent with the overall findings. These results underscore the need for further research focusing on specific stoma types to refine prophylactic strategies.

ES demonstrated a significant reduction in PSH risk compared to control groups, with efficacy comparable to AWSE but inferior to FM. While effective, ES pose technical challenges, particularly with laparoscopic approaches. Robotic assistance may facilitate their execution but increases operative times, which could lead to surgeon fatigue and reduced consistency in application. Similarly, despite its apparent efficacy, SMART is supported only by three observational studies [11, 28, 29], with no randomised trials available. This results in a low level of evidence for its recommendation. Additionally, its high resource utilization and technical complexity underscore the need to balance effectiveness with feasibility in diverse healthcare settings.

AWE proved to be a practical and effective alternative, showing comparable efficacy to FM, SMART and ES while surpassing other interventions such as sublay and IPM. Beyond their simplicity, utility and scalability, particularly attractive in resource-limited settings, the prophylactic effect of AWSE can be attributed to their role in enhancing core muscle strength and abdominal wall integrity. This dynamic support helps to more effectively distribute intra-abdominal pressure at the stoma site, potentially mitigating fascial weakening or enlargement of the parastomal defect.

However, the heterogeneity observed in the network meta-analysis likely reflects variability in how exercises were implemented across studies [13, 30]. It is crucial to highlight that outcomes appear strongly correlated with the quality and, particularly, the supervision of the exercise regimen [30, 31]. This underscores that unsupervised or poorly executed exercises may yield suboptimal results [13]. Furthermore, the concept of prehabilitation, involving targeted abdominal wall strengthening prior to surgery, has demonstrated significant promise in reducing recurrence rates for incisional hernias, suggesting a broader beneficial role for such exercises. Future research should thus prioritize standardized and supervised AWSE protocols to fully ascertain and optimize their prophylactic potential for PSH [90].

The findings align with prior meta-analyses that support the use of SM to reduce PSH risk, but they contrast with the conclusions of Verdaguer-Tremolosa et al. [91], who reported no benefit from prophylactic meshes. This discrepancy may stem from differences in methodology, including the failure to stratify mesh types and surgical techniques in previous analyses.

The subgroup analysis demonstrated the consistency and robustness of the results by confirming the protective role of the prophylactic measures identified in the initial analysis. In patients undergoing ileal conduit creation, the analysis highlighted ES and SM as the two most effective interventions for reducing PSH risk. However, these findings should be interpreted with caution because of the limited number of studies available in this subgroup, resulting in a low level of recommendation for these measures.

The sensitivity analysis restricted to studies using CT for PSH diagnosis confirmed the robustness of the main findings, supporting the efficacy of funnel mesh, SMART, exercises and extraperitoneal stomas regardless of the diagnostic approach.

Unlike prior studies, this network meta-analysis provides a comprehensive ranking of prophylactic measures for PSH prevention, integrating both direct and indirect evidence across diverse interventions. These findings represent a step forward in guiding individualized clinical decision-making.

However, certain limitations must be acknowledged. The inclusion of observational studies alongside RCTs introduced a higher risk of bias due to confounding and selection bias. A significant proportion of comparisons relied on indirect evidence, reducing the robustness of the conclusions. Heterogeneity was moderate to high, reflecting variability in study populations, surgical techniques and follow-up durations, which ranged from 6 to 60 months. Inconsistencies in outcome definitions and diagnostic criteria for PSH further complicate cross-study comparisons. Lastly, publication bias was detected, suggesting a potential overestimation of the efficacy of some interventions as a result of selective reporting.

Moderate-to-high heterogeneity observed in this meta-analysis reflects variability in study populations, surgical techniques and follow-up durations. Standardized protocols for reporting surgical techniques and outcomes are essential to improve the comparability of future studies and enhance the generalizability of findings.

On the basis of these findings, the authors recommend considering FM for high-risk patients with significant risk factors such as obesity, corticosteroid dependence or prior abdominal wall hernias. For most patients, simpler and equally effective interventions, such ES or AWSE, may provide optimal outcomes while minimizing complexity and resource use.

Future research should focus on conducting well-designed RCTs with standardized methodologies and outcome reporting to strengthen the evidence and improve clinical guidelines. Special attention should be given to assessing the long-term effectiveness of AWSE and determining the role of ES in PSH prevention.

## Conclusion

Prophylactic interventions with the most significant efficacy compared to control included funnel mesh, SMART technique, AWSE, extraperitoneal stoma creation and sublay mesh reinforcement. However, the degree of recommendation for each intervention varies considerably. This is due to the considerable heterogeneity observed in the studies included in the meta-analysis. The results suggest that, particularly in patients with multiple risk factors, funnel mesh implantation is likely to be the most effective prophylactic strategy.

## Supplementary Information

Below is the link to the electronic supplementary material.

Supplementary file1 (DOCX 496 kb)

Supplementary file2 (DOCX 160 kb)

Supplementary file3 (DOCX 29 kb)

Supplementary file4 (DOCX 33 kb)

## Data Availability

No datasets were generated or analysed during the current study.
