# Supplementary material for: Comparing the effectiveness of prophylactic strategies for parastomal hernia prevention: a network meta-analysis
Source: Tech Coloproctol. 2025 Sep 25;29(1):169. doi: 10.1007/s10151-025-03211-6 (PMC12464107; doi:10.1007/s10151-025-03211-6)
Supplement: Supplementary file 1 — Supplementary file1 (DOCX 496 kb) [file 10151_2025_3211_MOESM1_ESM.docx]

| **Prophylactic Measure** | **Technical Description** |
| --- | --- |
| **Transrectal stoma** | The stoma is directly externalized through anterior rectus abdominis muscle. |
| **Lateral Stoma** | Stoma placement lateral to the rectus sheath (7). |
| **Funnel Mesh** | The Funnel Mesh, also known as Hybrid 3D, is a 3D surgical mesh implant with a unique design. It is usually made from a synthetic material like PVDF (polyvinylidene fluoride), and it has a distinct funnel or cone shape (10). |
| **Sublay Mesh** | A synthetic mesh is strategically placed in a deep position relative to the rectus muscles, yet in a superficial position with respect to the posterior rectus sheath or peritoneum (i.e., in the preperitoneal or retromuscular space), enveloping the bowel loop as it traverses the abdominal wall to create the stoma (12). |
| **Intraperitoneal Mesh** | A synthetic or composite mesh is placed directly on the peritoneal surface, inside the abdominal cavity, over the opening created for the stoma. This mesh is usually anchored to the inner wall of the abdomen, enveloping or encircling the bowel segment as it leaves the body to create the stoma (9). |
| **Extraperitoneal Stoma** | The segment of bowel intended for the stoma is brought to the skin surface. This is done by dissecting through the abdominal wall layers. These layers are muscle and fascia. They are located outside the peritoneal cavity. The objective is to build a long, narrow, and well-supported tunnel for the bowel, as opposed to simply bringing it through a direct opening into the abdomen (8). |
| **SMART / STORMM** | A prophylactic surgical technique for parastomal hernias is used that utilizes a specialized circular stapling device to create the stoma trephine (abdominal wall opening) and simultaneously have a mesh circumferentially fixed around the stoma, often in a sub-peritoneal position, to reinforce the abdominal wall aperture and prevent future herniation (11). |
| **Abdominal Wall Exercises** | Patients participated in a trunk muscle strengthening program. The Rehabilitation Unit designed the program. Physiotherapists and stomatotherapists supervised it. The exercise regimen was started at least six weeks before the scheduled surgery (13). |

**Table 1.** Definitions of Surgical Techniques Used for Parastomal Hernia Prevention.


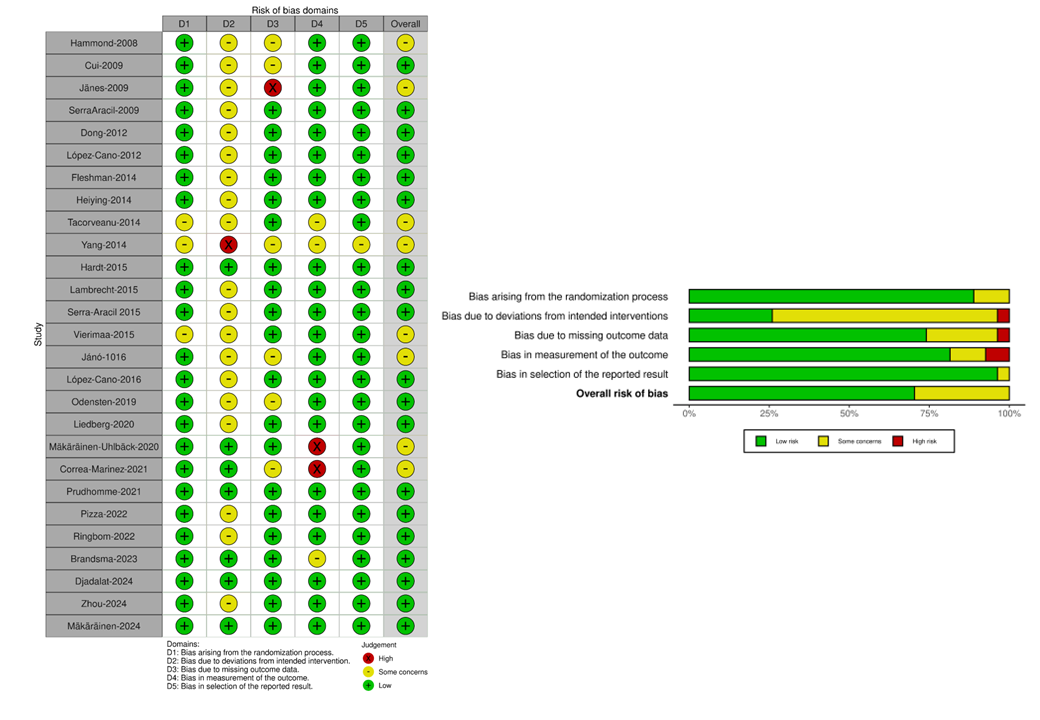


**Figure 2.** Quality of evidence for randomized clinical trials included in network meta-analysis.


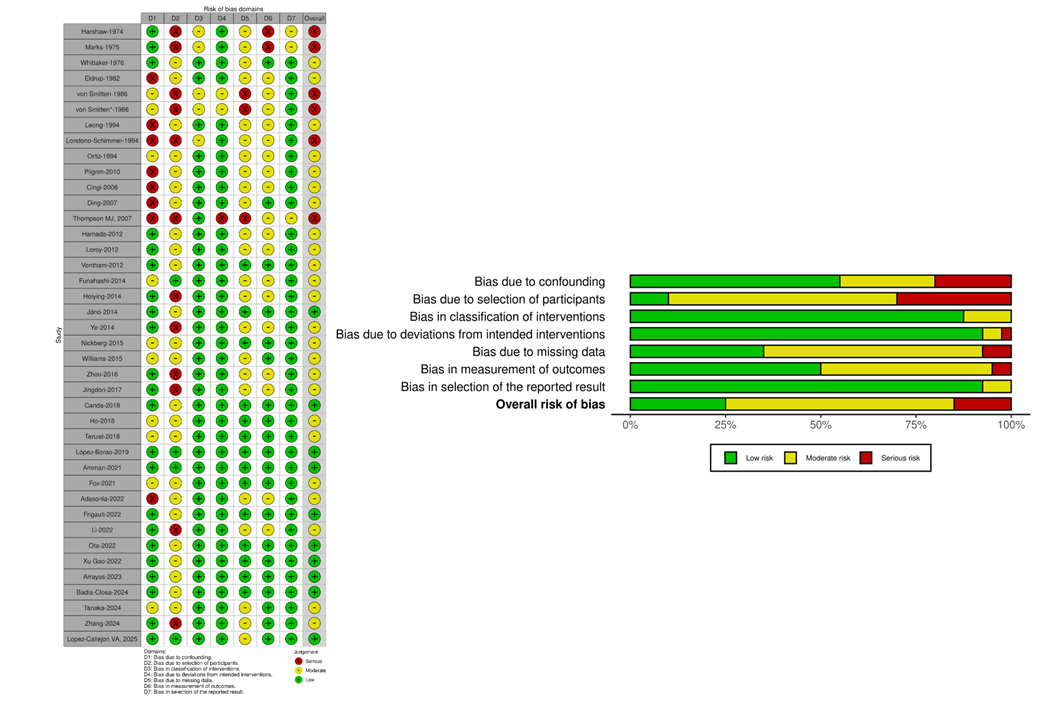


**Figure 3.** Quality of evidence for retrospective studies included in network meta-analysis.
