# Supplementary material for: Comparing the effectiveness of prophylactic strategies for parastomal hernia prevention: a network meta-analysis
Source: Tech Coloproctol. 2025 Sep 25;29(1):169. doi: 10.1007/s10151-025-03211-6 (PMC12464107; doi:10.1007/s10151-025-03211-6)
Supplement: Supplementary file 2 — Supplementary file2 (DOCX 160 kb) [file 10151_2025_3211_MOESM2_ESM.docx]

**
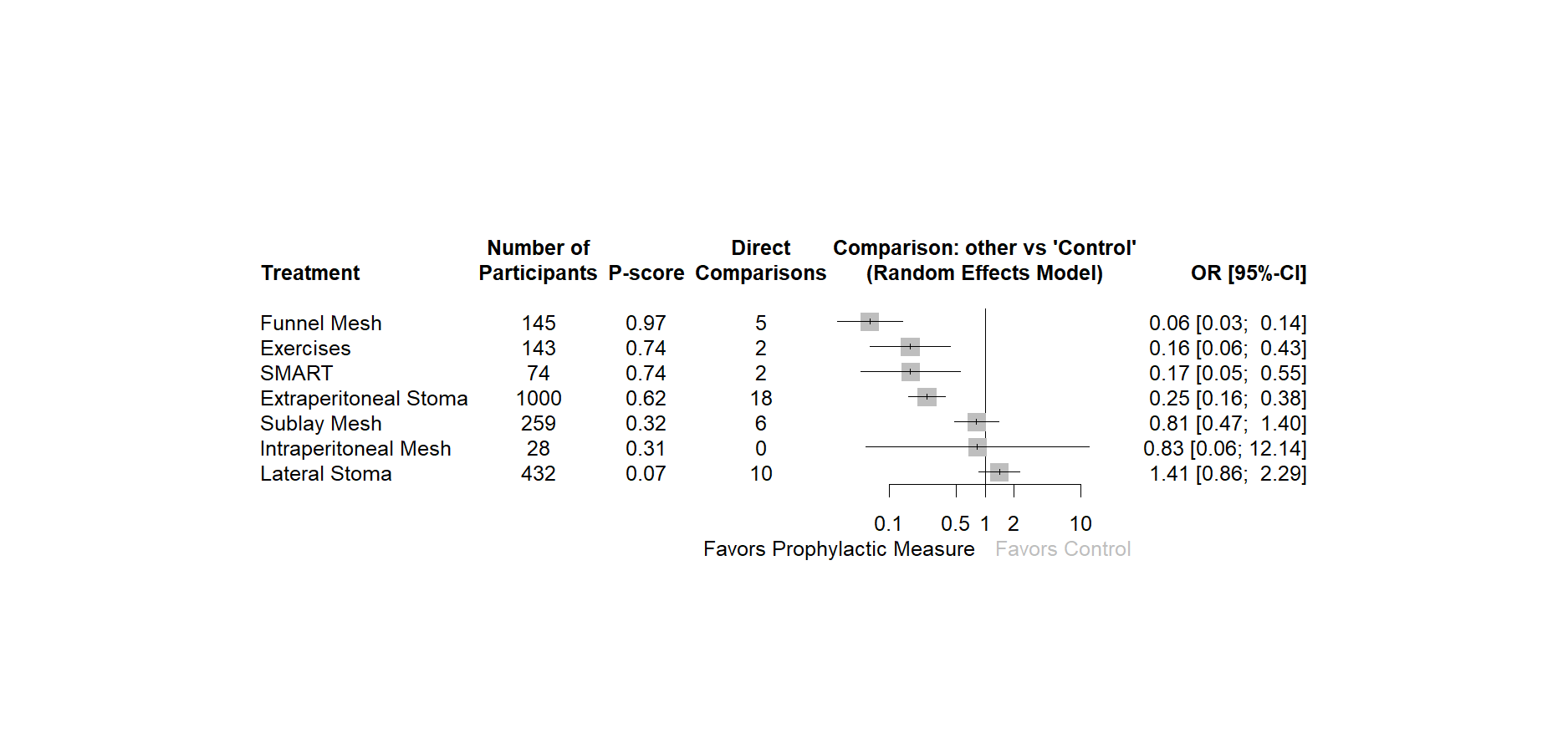

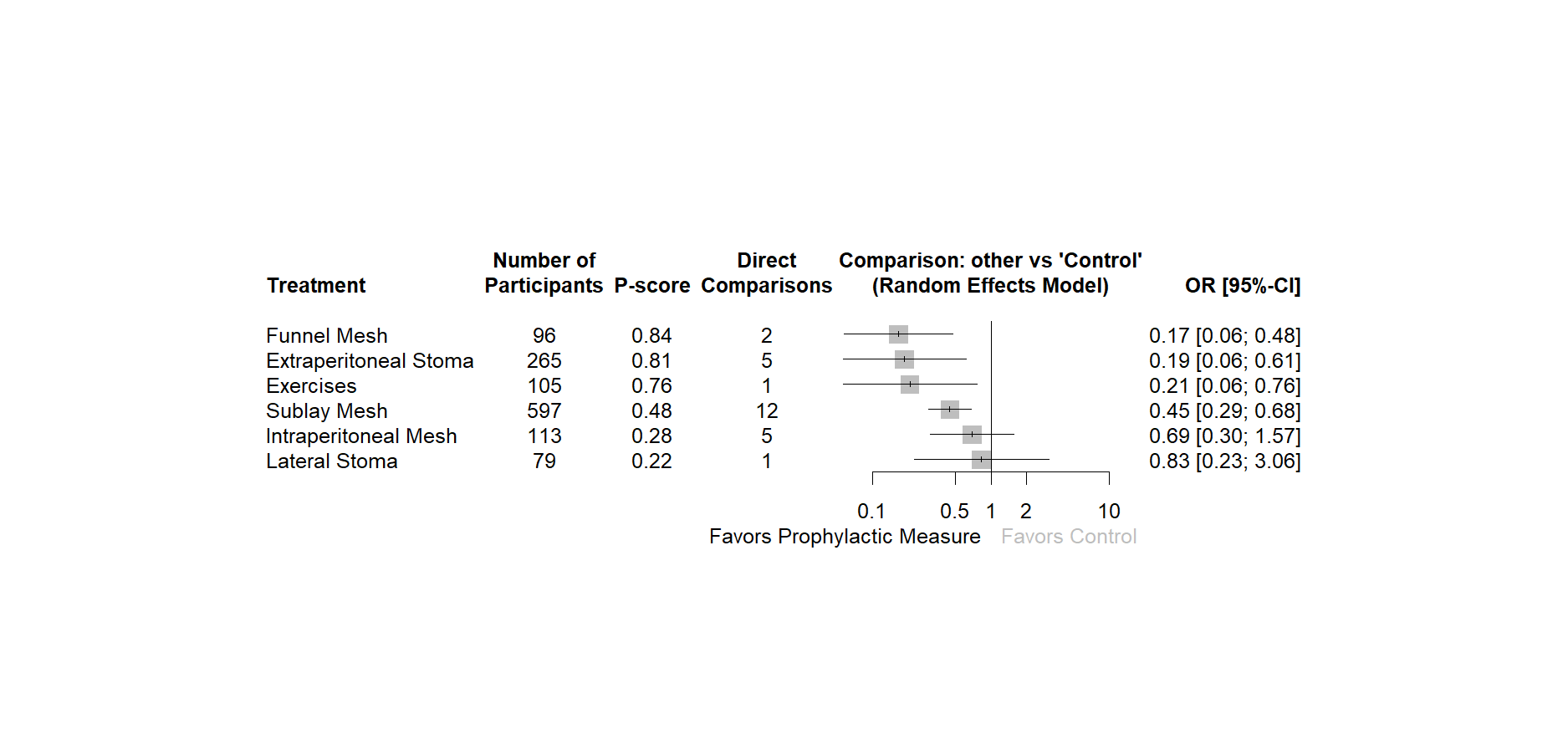
Figure 1**. Forest plot depicting the network model for Randomized Clinical Trials.

**Figure 2.** Forest plot depicting the network model for Non Randomized Clinical Trials.

**
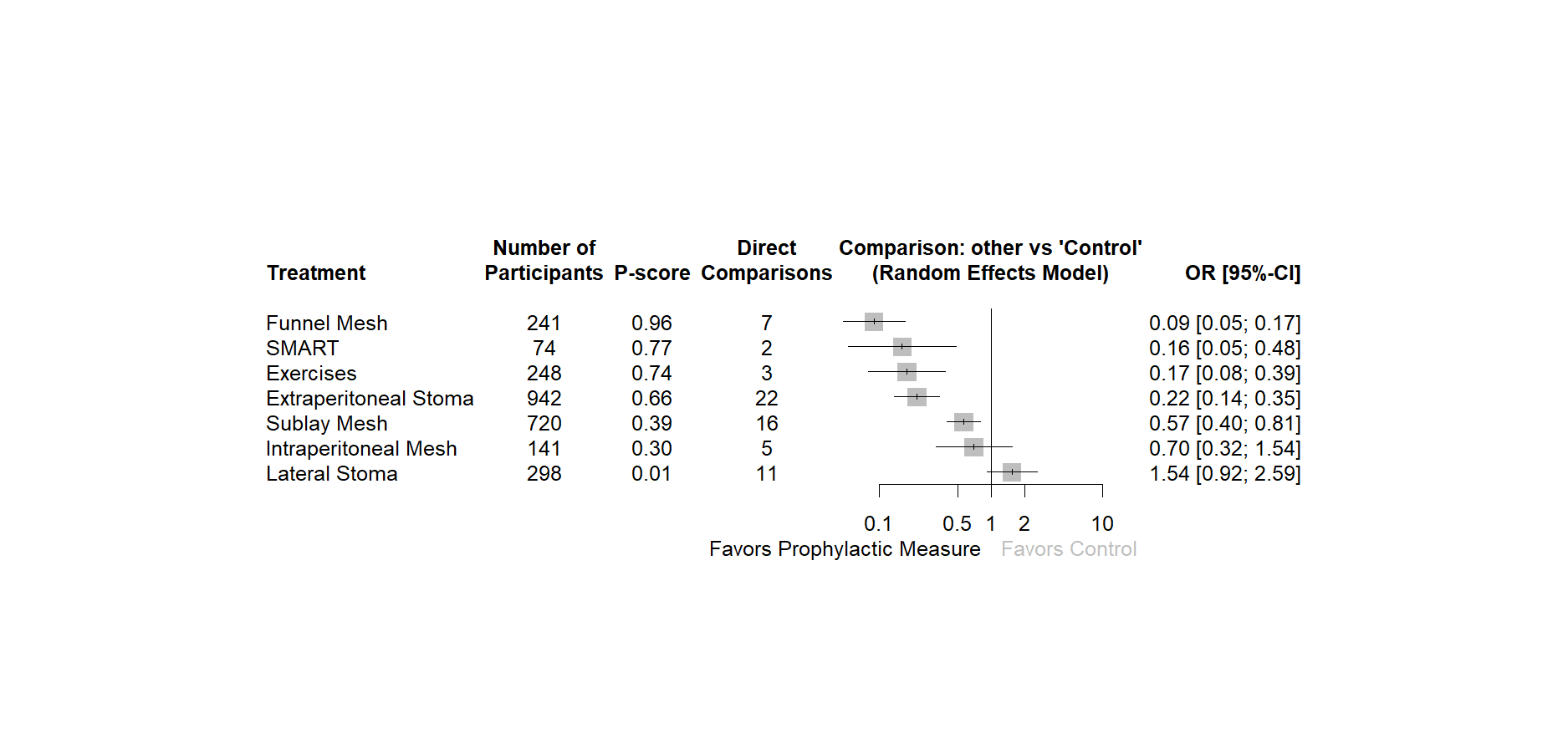
Figure 3**. Forest plot depicting the network model for digestive stomas.

**
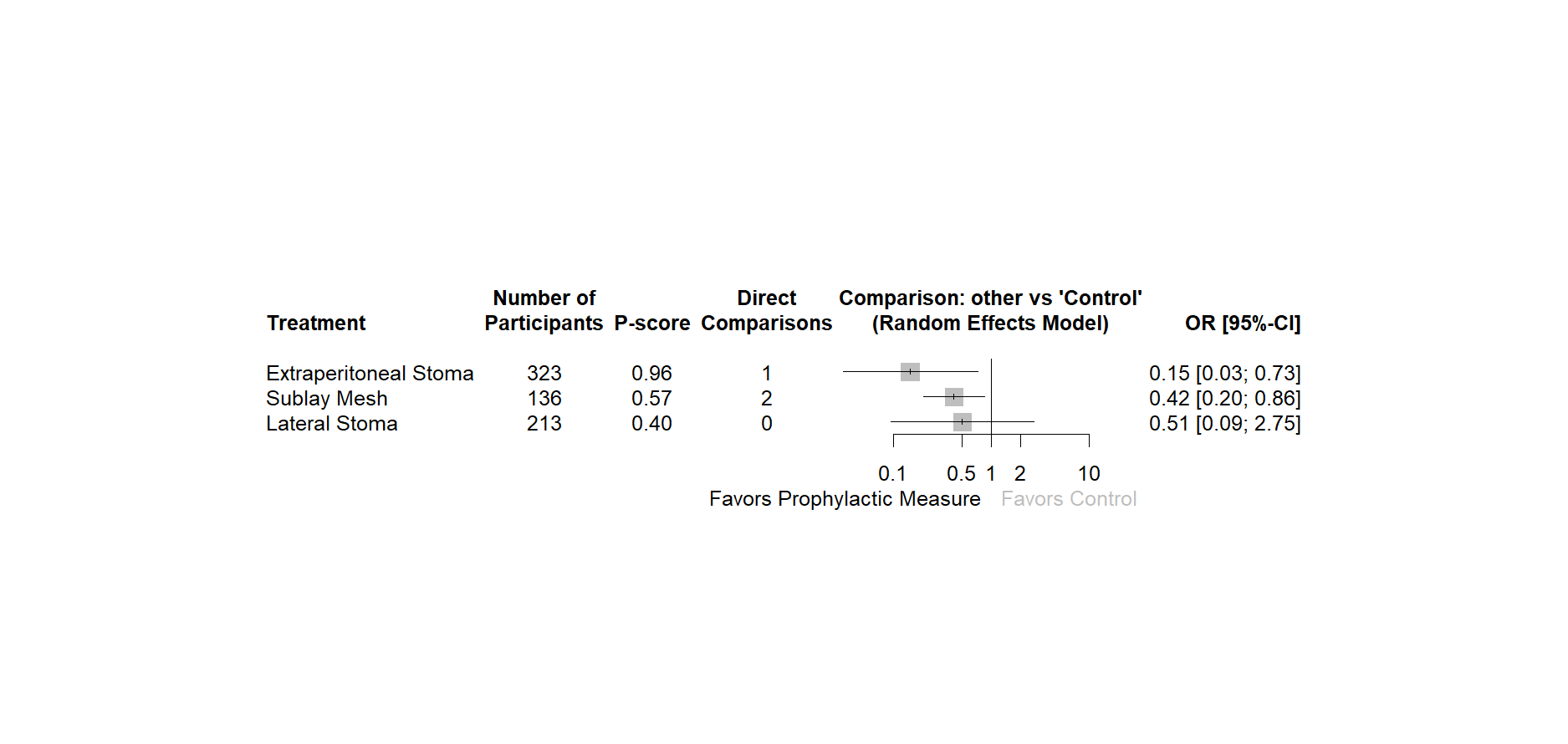
Figure 4.** Forest plot depicting the network model for ileal conduit.

**
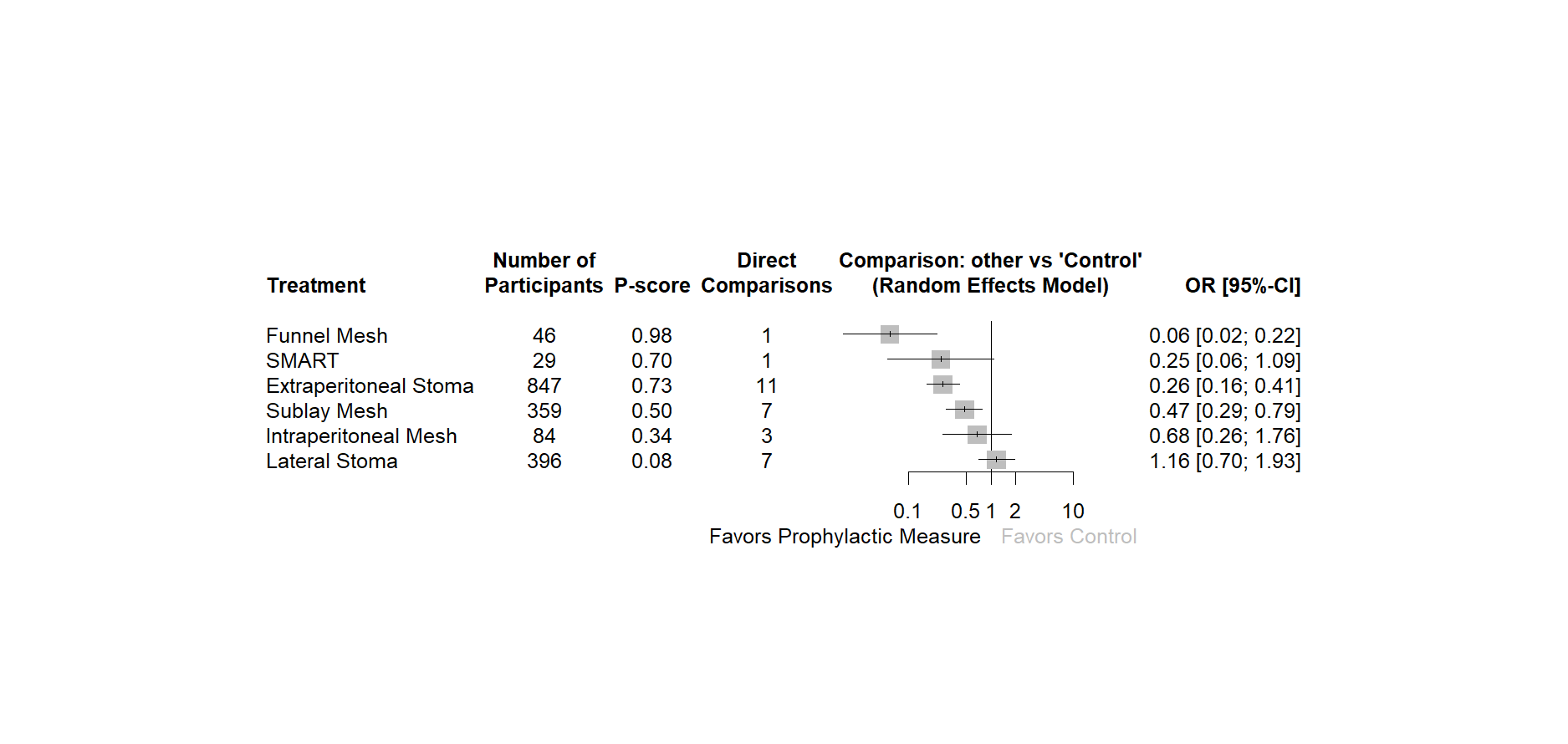
Figure 5.** Forest plot depicting the network model for follow-up ≥ 24 months.


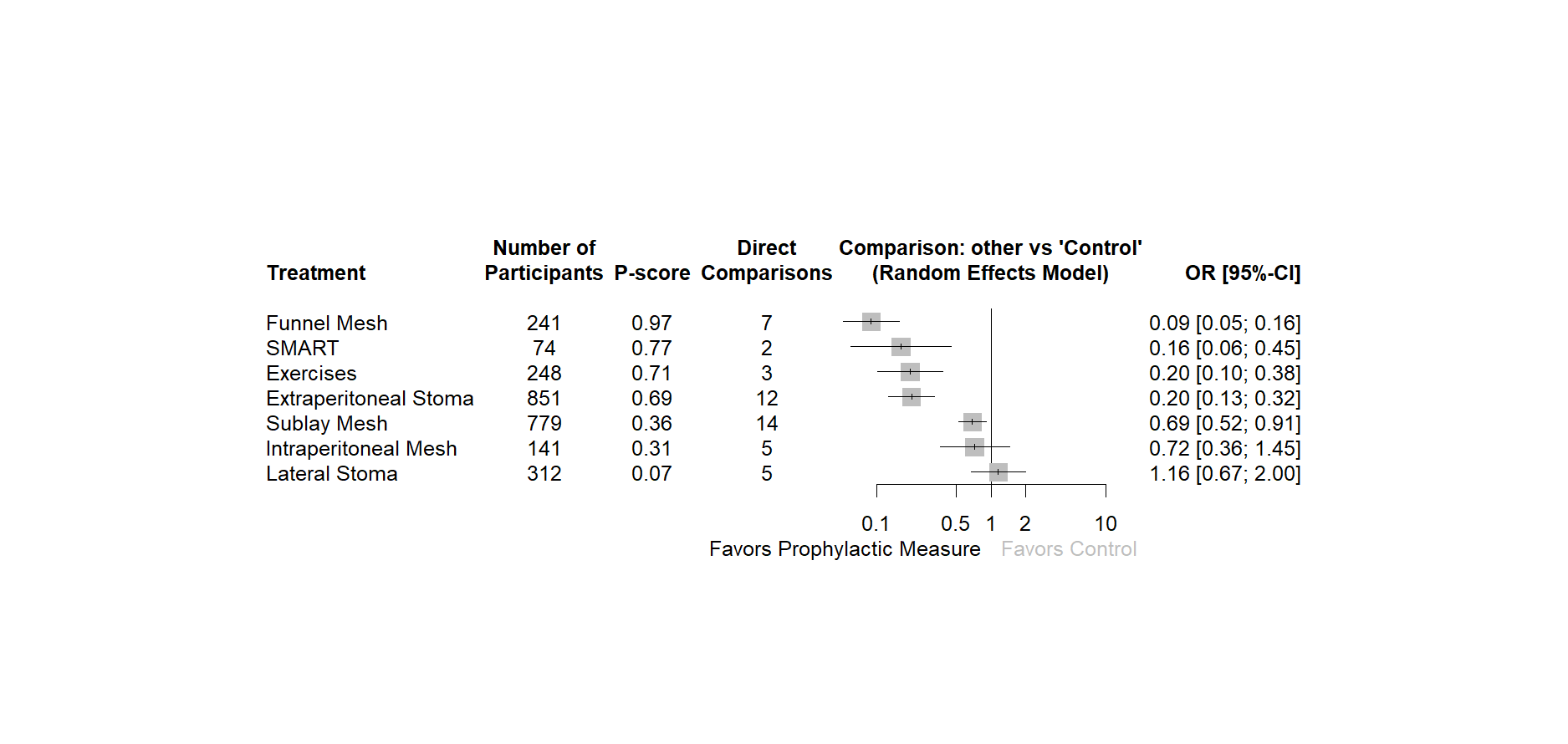


**Figure 6.** Forest plot depicting the model for Computed Tomography (CT) diagnosis of parastomal hernia.
