# Supplementary material for: Comparing the effectiveness of prophylactic strategies for parastomal hernia prevention: a network meta-analysis
Source: Tech Coloproctol. 2025 Sep 25;29(1):169. doi: 10.1007/s10151-025-03211-6 (PMC12464107; doi:10.1007/s10151-025-03211-6)
Supplement: Supplementary file 3 — Supplementary file3 (DOCX 29 kb) [file 10151_2025_3211_MOESM3_ESM.docx]

| **Certainty assessment** | | | | | | | **№ of patients** | | **Effect** | | **Certainty** | **Importance** |
| --- | --- | --- | --- | --- | --- | --- | --- | --- | --- | --- | --- | --- |
| **№ of studies** | **Study design** | **Risk of bias** | **Inconsistency** | **Indirectness** | **Imprecision** | **Other considerations** | **Funnel Mesh use** | **Control** | **Relative (95% CI)** | **Absolute (95% CI)** |  |  |
| **Parastomal Hernia (RCT) (follow-up: median 15.5 months; assessed with: Number)** | | | | | | | | | | | | |
| 2 | randomised trials | not serious | not serious | not serious | not serious | strong association | 9/96 (9.4%) | 40/105 (38.1%) | **OR 0.17** (0.06 to 0.48) | **286 fewer per 1000** (from 345 fewer to 153 fewer) | ⨁⨁⨁⨁ High | CRITICAL |
| **Parastomal Hernia (Retrospective studies) (follow-up: median 15 months; assessed with: Number)** | | | | | | | | | | | | |
| 5 | non-randomised studies | serious^a,b,c^ | not serious | not serious | not serious | strong association all plausible residual confounding would reduce the demonstrated effect | 13/145 (9.0%) | 117/199 (58.8%) | **OR 0.06** (0.03 to 0.14) | **509 fewer per 1000** (from 547 fewer to 421 fewer) | ⨁⨁⨁◯ Moderate^a,b,c^ | IMPORTANT |

**CI:** confidence interval; **OR:** odds ratio

#### Explanations

a. Retrospective studies

b. Different strategies to diagnose PSH

c. Possible bias selection

Table 1. Funnel Mesh vs Control for parastomal hernia prophylaxis.

| **Certainty assessment** | | | | | | | **№ of patients** | | **Effect** | | **Certainty** | **Importance** |
| --- | --- | --- | --- | --- | --- | --- | --- | --- | --- | --- | --- | --- |
| **№ of studies** | **Study design** | **Risk of bias** | **Inconsistency** | **Indirectness** | **Imprecision** | **Other considerations** | **SMART-STORMM Technique** | **Control** | **Relative (95% CI)** | **Absolute (95% CI)** |  |  |
| **Parastomal Hernia (Retrospective studies) (follow-up: median 24 months; assessed with: Number)** | | | | | | | | | | | | |
| 2 | non-randomised studies | serious^a,b,c^ | not serious | not serious | not serious | all plausible residual confounding would reduce the demonstrated effect | 8/50 (16.0%) | 23/49 (46.9%) | **OR 0.17** (0.05 to 0.55) | **339 fewer per 1000** (from 427 fewer to 142 fewer) | ⨁⨁◯◯ Low^a,b^ | IMPORTANT |

**CI:** confidence interval; **OR:** odds ratio

#### Explanations

a. Follow-up limited

b. Surgical techniques differencies

c. Possible selection bias

Table 2. SMART-STORMM vs Control for parastomal hernia prophylaxis.

| **Certainty assessment** | | | | | | | **№ of patients** | | **Effect** | | **Certainty** | **Importance** |
| --- | --- | --- | --- | --- | --- | --- | --- | --- | --- | --- | --- | --- |
| **№ of studies** | **Study design** | **Risk of bias** | **Inconsistency** | **Indirectness** | **Imprecision** | **Other considerations** | **Abdominal training exercises** | **Control** | **Relative (95% CI)** | **Absolute (95% CI)** |  |  |
| **Parastomal Hernia (follow-up: median 6 months; assessed with: Number)** | | | | | | | | | | | | |
| 1 | randomised trials | serious^a^ | not serious | not serious | not serious | strong association all plausible residual confounding would reduce the demonstrated effect | 7/105 (6.7%) | 27/105 (25.7%) | **OR 0.21** (0.06 to 0.76) | **189 fewer per 1000** (from 237 fewer to 49 fewer) | ⨁⨁⨁⨁ High^a^ | IMPORTANT |
| **Parastomal Hernia (follow-up: median 12 months; assessed with: Number)** | | | | | | | | | | | | |
| 2 | non-randomised studies | serious^b,c^ | not serious | not serious | not serious | strong association all plausible residual confounding would reduce the demonstrated effect | 20/143 (14.0%) | 57/123 (46.3%) | **OR 0.16** (0.06 to 0.43) | **342 fewer per 1000** (from 414 fewer to 193 fewer) | ⨁⨁⨁◯ Moderate^b,c^ | CRITICAL |

**CI:** confidence interval; **OR:** odds ratio

#### Explanations

a. Follow-up: 6 months

b. Risk of selection bias

c. Limited follow-up

Table 3. Exercises vs Control for parastomal hernia prophylaxis.

| **Certainty assessment** | | | | | | | **№ of patients** | | **Effect** | | **Certainty** | **Importance** |
| --- | --- | --- | --- | --- | --- | --- | --- | --- | --- | --- | --- | --- |
| **№ of studies** | **Study design** | **Risk of bias** | **Inconsistency** | **Indirectness** | **Imprecision** | **Other considerations** | **Extraperitoneal route of stoma** | **Control** | **Relative (95% CI)** | **Absolute (95% CI)** |  |  |
| **Parastomal Hernia (follow-up: median 12 months; assessed with: Number)** | | | | | | | | | | | | |
| 5 | randomised trials | not serious | not serious | not serious | not serious | strong association | 0/213 (0.0%) | 15/207 (7.2%) | **OR 0.19** (0.06 to 0.61) | **58 fewer per 1000** (from 68 fewer to 27 fewer) | ⨁⨁⨁⨁ High | CRITICAL |
| **Parastomal Hernia (assessed with: Number)** | | | | | | | | | | | | |
| 18 | non-randomised studies | serious^a,b,c^ | not serious | not serious | not serious | strong association all plausible residual confounding would reduce the demonstrated effect | 55/1028 (5.4%) | 278/1507 (18.4%) | **OR 0.25** (0.16 to 0.38) | **131 fewer per 1000** (from 150 fewer to 105 fewer) | ⨁⨁⨁◯ Moderate^a,b,c^ | CRITICAL |

**CI:** confidence interval; **OR:** odds ratio

#### Explanations

a. The diagnosis of PSH was clinical only in several studies.

b. Follow-up was very heterogeneous.

c. Possible selection bias

Table 4. Extraperitoneal route of stoma vs Control for parastomal hernia prophylaxis.

| **Certainty assessment** | | | | | | | **№ of patients** | | **Effect** | | **Certainty** | **Importance** |
| --- | --- | --- | --- | --- | --- | --- | --- | --- | --- | --- | --- | --- |
| **№ of studies** | **Study design** | **Risk of bias** | **Inconsistency** | **Indirectness** | **Imprecision** | **Other considerations** | **Sublay Mesh** | **Control** | **Relative (95% CI)** | **Absolute (95% CI)** |  |  |
| **Parastomal Hernia (follow-up: median 24 months; assessed with: Number)** | | | | | | | | | | | | |
| 14 | randomised trials | serious^a,b^ | not serious | not serious | serious^c^ | strong association all plausible residual confounding would reduce the demonstrated effect | 154/597 (25.8%) | 280/724 (38.7%) | **OR 0.45** (0.29 to 0.68) | **166 fewer per 1000** (from 232 fewer to 87 fewer) | ⨁⨁⨁⨁ High^a,b,c^ | IMPORTANT |
| **Parastomal Hernia (Retrospective studies) (follow-up: median 20.5 months; assessed with: Number)** | | | | | | | | | | | | |
| 6 | non-randomised studies | serious^a,b,d,e,f^ | not serious | not serious | not serious | all plausible residual confounding would reduce the demonstrated effect^g^ | 121/259 (46.7%) | 183/368 (49.7%) | **OR 0.81** (0.47 to 1.40) | **52 fewer per 1000** (from 180 fewer to 83 more) | ⨁⨁◯◯ Low^a,b,d,e,f,g^ | NOT IMPORTANT |

**CI:** confidence interval; **OR:** odds ratio

#### Explanations

a. Highly heterogeneous follow-ups in the series

b. Different types of chemical composition of the meshes used.

c. Colostomy and ileal conduit are inclosed in meta-analysis

d. Surgical approach was very heterogeneous with a mix of open, laparoscopic and robotic approach.

e. Only clinical diagnosis was performed in a significative number of studies.

f. Egger's Test demostrated significant statistical differences in publications.

Table 5. Sublay Mesh vs Control for parastomal hernia prophylaxis.

| **Certainty assessment** | | | | | | | **№ of patients** | | **Effect** | | **Certainty** | **Importance** |
| --- | --- | --- | --- | --- | --- | --- | --- | --- | --- | --- | --- | --- |
| **№ of studies** | **Study design** | **Risk of bias** | **Inconsistency** | **Indirectness** | **Imprecision** | **Other considerations** | **Intraperitoneal Mesh** | **Control** | **Relative (95% CI)** | **Absolute (95% CI)** |  |  |
| **Parastomal Hernia (RCT) (follow-up: median 17; assessed with: Number)** | | | | | | | | | | | | |
| 5 | randomised trials | serious^a,b^ | not serious | not serious | not serious | none | 48/113 (42.5%) | 69/125 (55.2%) | **OR 0.69** (0.30 to 1.57) | **92 fewer per 1000** (from 282 fewer to 107 more) | ⨁⨁⨁◯ Moderate^a,b^ | IMPORTANT |

**CI:** confidence interval; **OR:** odds ratio

#### Explanations

a. Different surgical techniques were applied.

b. Surgical approach mixed

Table 6. Intraperitoneal Mesh vs Control for parastomal hernia prophylaxis.

| **Certainty assessment** | | | | | | | **№ of patients** | | **Effect** | | **Certainty** | **Importance** |
| --- | --- | --- | --- | --- | --- | --- | --- | --- | --- | --- | --- | --- |
| **№ of studies** | **Study design** | **Risk of bias** | **Inconsistency** | **Indirectness** | **Imprecision** | **Other considerations** | **Lateral Stoma** | **Control** | **Relative (95% CI)** | **Absolute (95% CI)** |  |  |
| **Parastomal hernia (RCT) (follow-up: median 118 months; assessed with: Number)** | | | | | | | | | | | | |
| 1 | randomised trials | not serious | not serious | not serious | not serious | none | 5/27 (18.5%) | 4/29 (13.8%) | **OR 0.83** (0.23 to 3.06) | **21 fewer per 1000** (from 102 fewer to 191 more) | ⨁⨁⨁⨁ High | NOT IMPORTANT |
| **Parastomal hernia (Retrospective studies) (follow-up: median 24 months; assessed with: Number)** | | | | | | | | | | | | |
| 10 | non-randomised studies | serious^a,b,c^ | not serious | not serious | not serious^a^ | all plausible residual confounding would reduce the demonstrated effect | 89/240 (37.1%) | 170/571 (29.8%) | **OR 1.41** (0.86 to 2.29) | **76 more per 1000** (from 31 fewer to 195 more) | ⨁⨁◯◯ Low^a^ | NOT IMPORTANT |

**CI:** confidence interval; **OR:** odds ratio

#### Explanations

1. Mixed surgical approach
2. Mixed type of stoma
3. Heterogeneous follow-up
4. Possible selection bias

Table 7. Lateral stoma vs Control for parastomal hernia prophylaxis.
