# Supplementary material for: Comparing the effectiveness of prophylactic strategies for parastomal hernia prevention: a network meta-analysis
Source: Tech Coloproctol. 2025 Sep 25;29(1):169. doi: 10.1007/s10151-025-03211-6 (PMC12464107; doi:10.1007/s10151-025-03211-6)
Supplement: Supplementary file 4 — Supplementary file4 (DOCX 33 kb) [file 10151_2025_3211_MOESM4_ESM.docx]

|  | | | | | | |
| --- | --- | --- | --- | --- | --- | --- |
| **Funnel Mesh use compared to Control for Parastomal Hernia Prophylaxis** | | | | | | |
|  | | | | | | |
| Outcomes | **Anticipated absolute effects^*^** (95% CI) | | Relative effect (95% CI) | № of participants (studies) | Certainty of the evidence (GRADE) | Comments |
|  | **Risk with Control** | **Risk with Funnel Mesh use** |  |  |  |  |
| Parastomal Hernia (RCT) (PSH) assessed with: Number follow-up: median 15.5 months | 381 per 1000 | **95 per 1000** (36 to 228) | **OR 0.17** (0.06 to 0.48) | 201 (2 RCTs) | ⨁⨁⨁⨁ High | 3D Mesh use results in large reduction in parastomal Hernia. |
| Parastomal Hernia (Retrospective studies) (PSH) assessed with: Number follow-up: median 15 months | 588 per 1000 | **79 per 1000** (41 to 166) | **OR 0.06** (0.03 to 0.14) | 344 (5 non-randomised studies) | ⨁⨁⨁◯ Moderate^a,b,c^ | 3D Mesh use likely reduces parastomal Hernia (Retrospective studies). |
| ***The risk in the intervention group** (and its 95% confidence interval) is based on the assumed risk in the comparison group and the **relative effect** of the intervention (and its 95% CI).  **CI:** confidence interval; **OR:** odds ratio | | | | | | |
| **GRADE Working Group grades of evidence** **High certainty:** we are very confident that the true effect lies close to that of the estimate of the effect. **Moderate certainty:** we are moderately confident in the effect estimate: the true effect is likely to be close to the estimate of the effect, but there is a possibility that it is substantially different. **Low certainty:** our confidence in the effect estimate is limited: the true effect may be substantially different from the estimate of the effect. **Very low certainty:** we have very little confidence in the effect estimate: the true effect is likely to be substantially different from the estimate of effect. | | | | | | |

#### Explanations

a. Retrospective studies

b. Different strategies to diagnose PSH

c. Possible selection bias

Table 1. Summary of Funnel Mesh compared to Control for Parastomal Hernia Prophylaxis

|  | | | | | | |
| --- | --- | --- | --- | --- | --- | --- |
| **SMART-STORMM Technique compared to Control for Parastomal Hernia Prophylaxis** | | | | | | |
|  | | | | | | |
| Outcomes | **Anticipated absolute effects^*^** (95% CI) | | Relative effect (95% CI) | № of participants (studies) | Certainty of the evidence (GRADE) | Comments |
|  | **Risk with Control** | **Risk with SMART-STORMM Technique** |  |  |  |  |
| Parastomal Hernia (Retrospective studies) (PSH) assessed with: Number follow-up: median 24 months | 469 per 1000 | **131 per 1000** (42 to 327) | **OR 0.17** (0.05 to 0.55) | 99 (2 non-randomised studies) | ⨁⨁◯◯ Low^a,b^ | SMART-STORMM Technique may result in a large reduction in parastomal Hernia (Retrospective studies). |
| ***The risk in the intervention group** (and its 95% confidence interval) is based on the assumed risk in the comparison group and the **relative effect** of the intervention (and its 95% CI).  **CI:** confidence interval; **OR:** odds ratio | | | | | | |
| **GRADE Working Group grades of evidence** **High certainty:** we are very confident that the true effect lies close to that of the estimate of the effect. **Moderate certainty:** we are moderately confident in the effect estimate: the true effect is likely to be close to the estimate of the effect, but there is a possibility that it is substantially different. **Low certainty:** our confidence in the effect estimate is limited: the true effect may be substantially different from the estimate of the effect. **Very low certainty:** we have very little confidence in the effect estimate: the true effect is likely to be substantially different from the estimate of effect. | | | | | | |

#### Explanations

a. Follow-up limited

b. Surgical techniques differencies

c. Possible selection bias

Table 2. Summary of SMART-STORMM Technique compared to Control for Parastomal Hernia Prophylaxis

|  | | | | | | |
| --- | --- | --- | --- | --- | --- | --- |
| **Abdominal training exercises compared to Control for Parastomal Hernia Prophylaxis** | | | | | | |
|  | | | | | | |
| Outcomes | **Anticipated absolute effects^*^** (95% CI) | | Relative effect (95% CI) | № of participants (studies) | Certainty of the evidence (GRADE) | Comments |
|  | **Risk with Control** | **Risk with Abdominal training exercises** |  |  |  |  |
| Parastomal Hernia (PSH) assessed with: Number follow-up: median 6 months | 257 per 1000 | **68 per 1000** (20 to 208) | **OR 0.21** (0.06 to 0.76) | 210 (1 RCT) | ⨁⨁⨁⨁ High^a^ | Abdominal training exercises results in a reduction in parastomal Hernia. |
| Parastomal Hernia (PSH) assessed with: Number follow-up: median 12 months | 463 per 1000 | **121 per 1000** (49 to 271) | **OR 0.16** (0.06 to 0.43) | 266 (2 non-randomised studies) | ⨁⨁⨁◯ Moderate^b,c,d^ | Abdominal training exercises likely reduces parastomal Hernia. |
| ***The risk in the intervention group** (and its 95% confidence interval) is based on the assumed risk in the comparison group and the **relative effect** of the intervention (and its 95% CI).  **CI:** confidence interval; **OR:** odds ratio | | | | | | |
| **GRADE Working Group grades of evidence** **High certainty:** we are very confident that the true effect lies close to that of the estimate of the effect. **Moderate certainty:** we are moderately confident in the effect estimate: the true effect is likely to be close to the estimate of the effect, but there is a possibility that it is substantially different. **Low certainty:** our confidence in the effect estimate is limited: the true effect may be substantially different from the estimate of the effect. **Very low certainty:** we have very little confidence in the effect estimate: the true effect is likely to be substantially different from the estimate of effect. | | | | | | |

#### Explanations

a. Follow-up: 6 months

b. Risk of selection bias

c. Limited follow-up

d. Possible selection bias

Table 3. Summary of abdominal training exercises compared to Control for Parastomal Hernia Prophylaxis

|  | | | | | | |
| --- | --- | --- | --- | --- | --- | --- |
| **Extraperitoneal route of stoma compared to Control for Parastomal Hernia Prophylaxis** | | | | | | |
|  | | | | | | |
| Outcomes | **Anticipated absolute effects^*^** (95% CI) | | Relative effect (95% CI) | № of participants (studies) | Certainty of the evidence (GRADE) | Comments |
|  | **Risk with Control** | **Risk with Extraperitoneal route of stoma** |  |  |  |  |
| Parastomal Hernia (PSH) assessed with: Number follow-up: median 12 months | 72 per 1000 | **15 per 1000** (5 to 45) | **OR 0.19** (0.06 to 0.61) | 420 (5 RCTs) | ⨁⨁⨁⨁ High | Extraperitoneal route of stoma results in large reduction in parastomal Hernia. |
| Parastomal Hernia (PSH) assessed with: Number | 184 per 1000 | **54 per 1000** (35 to 79) | **OR 0.25** (0.16 to 0.38) | 2535 (18 non-randomised studies) | ⨁⨁⨁◯ Moderate^a,b,c^ | Extraperitoneal route of stoma likely results in a large reduction in parastomal Hernia. |
| ***The risk in the intervention group** (and its 95% confidence interval) is based on the assumed risk in the comparison group and the **relative effect** of the intervention (and its 95% CI).  **CI:** confidence interval; **OR:** odds ratio | | | | | | |
| **GRADE Working Group grades of evidence** **High certainty:** we are very confident that the true effect lies close to that of the estimate of the effect. **Moderate certainty:** we are moderately confident in the effect estimate: the true effect is likely to be close to the estimate of the effect, but there is a possibility that it is substantially different. **Low certainty:** our confidence in the effect estimate is limited: the true effect may be substantially different from the estimate of the effect. **Very low certainty:** we have very little confidence in the effect estimate: the true effect is likely to be substantially different from the estimate of effect. | | | | | | |

#### Explanations

a. The diagnosis of PSH was clinical only in several studies.

b. Follow-up was very heterogeneous.

c. Possible selection bias

Table 4. Summary of Extraperitoneal route of stoma compared to Control for Parastomal Hernia Prophylaxis

|  | | | | | | |
| --- | --- | --- | --- | --- | --- | --- |
| **Sublay Mesh compared to Control for Parastomal Hernia Prophylaxis** | | | | | | |
|  | | | | | | |
| Outcomes | **Anticipated absolute effects^*^** (95% CI) | | Relative effect (95% CI) | № of participants (studies) | Certainty of the evidence (GRADE) | Comments |
|  | **Risk with Control** | **Risk with Sublay Mesh** |  |  |  |  |
| Parastomal Hernia (PSH) assessed with: Number follow-up: median 24 months | 387 per 1000 | **221 per 1000** (155 to 300) | **OR 0.45** (0.29 to 0.68) | 1321 (14 RCTs) | ⨁⨁⨁⨁ High^a,b,c^ | Sublay Mesh results in large reduction in parastomal Hernia vs Control |
| Parastomal Hernia (Retrospective studies) (PSH) assessed with: Number follow-up: median 20.5 months | 497 per 1000 | **445 per 1000** (317 to 581) | **OR 0.81** (0.47 to 1.40) | 627 (6 non-randomised studies) | ⨁⨁◯◯ Low^a,b,d,e,f,^ | Sublay Mesh may reduce parastomal Hernia slightly vs Control (Retrospective studies) |
| ***The risk in the intervention group** (and its 95% confidence interval) is based on the assumed risk in the comparison group and the **relative effect** of the intervention (and its 95% CI).  **CI:** confidence interval; **OR:** odds ratio | | | | | | |
| **GRADE Working Group grades of evidence** **High certainty:** we are very confident that the true effect lies close to that of the estimate of the effect. **Moderate certainty:** we are moderately confident in the effect estimate: the true effect is likely to be close to the estimate of the effect, but there is a possibility that it is substantially different. **Low certainty:** our confidence in the effect estimate is limited: the true effect may be substantially different from the estimate of the effect. **Very low certainty:** we have very little confidence in the effect estimate: the true effect is likely to be substantially different from the estimate of effect. | | | | | | |

#### Explanations

a. Highly heterogeneous follow-ups in the series

b. Different types of chemical composition of the meshes used.

c. Colostomy and ileal conduit are inclosed in meta-analysis

d. Surgical approach was very heterogeneous with a mix of open, laparoscopic and robotic approach.

e. Only clinical diagnosis was performed in a significative number of studies.

f. Egger's Test demostrated significant statistical differences in publications.

Table 5. Summary of Sublay Mesh compared to Control for Parastomal Hernia Prophylaxis

|  | | | | | | |
| --- | --- | --- | --- | --- | --- | --- |
| **Intraperitoneal Mesh compared to Control for Parastomal Hernia Prophylaxis** | | | | | | |
|  | | | | | | |
| Outcomes | **Anticipated absolute effects^*^** (95% CI) | | Relative effect (95% CI) | № of participants (studies) | Certainty of the evidence (GRADE) | Comments |
|  | **Risk with Control** | **Risk with Intraperitoneal Mesh** |  |  |  |  |
| Parastomal Hernia (RCT) (PSH) assessed with: Number follow-up: median 17 | 552 per 1000 | **460 per 1000** (270 to 659) | **OR 0.69** (0.30 to 1.57) | 238 (5 RCTs) | ⨁⨁⨁◯ Moderate^a,b^ | Intraperitoneal Mesh likely results in a slight reduction in parastomal Hernia. |
| ***The risk in the intervention group** (and its 95% confidence interval) is based on the assumed risk in the comparison group and the **relative effect** of the intervention (and its 95% CI).  **CI:** confidence interval; **OR:** odds ratio | | | | | | |
| **GRADE Working Group grades of evidence** **High certainty:** we are very confident that the true effect lies close to that of the estimate of the effect. **Moderate certainty:** we are moderately confident in the effect estimate: the true effect is likely to be close to the estimate of the effect, but there is a possibility that it is substantially different. **Low certainty:** our confidence in the effect estimate is limited: the true effect may be substantially different from the estimate of the effect. **Very low certainty:** we have very little confidence in the effect estimate: the true effect is likely to be substantially different from the estimate of effect. | | | | | | |

#### Explanations

a. Different surgical techniques were applied.

b. Surgical approach mixed

Table 6. Summary of Intraperitoneal Mesh compared to Control for Parastomal Hernia Prophylaxis

|  | | | | | | |
| --- | --- | --- | --- | --- | --- | --- |
| **Lateral Stoma compared to Control for Parastomal Hernia Prophylaxis** | | | | | | |
|  | | | | | | |
| Outcomes | **Anticipated absolute effects^*^** (95% CI) | | Relative effect (95% CI) | № of participants (studies) | Certainty of the evidence (GRADE) | Comments |
|  | **Risk with Control** | **Risk with Lateral Stoma** |  |  |  |  |
| Parastomal hernia (RCT) (PSH) assessed with: Number follow-up: median 118 months | 138 per 1000 | **117 per 1000** (35 to 329) | **OR 0.83** (0.23 to 3.06) | 56 (1 RCT) | ⨁⨁⨁⨁ High | Lateral Stoma does not reduce parastomal hernia. |
| Parastomal hernia (Retrospective studies) (PSH) assessed with: Number follow-up: median 24 months | 298 per 1000 | **374 per 1000** (267 to 493) | **OR 1.41** (0.86 to 2.29) | 811 (10 non-randomised studies) | ⨁⨁◯◯ Low^a^ | Lateral Stoma may result in little to no difference in parastomal hernia (Retrospective studies). |
| ***The risk in the intervention group** (and its 95% confidence interval) is based on the assumed risk in the comparison group and the **relative effect** of the intervention (and its 95% CI).  **CI:** confidence interval; **OR:** odds ratio | | | | | | |
| **GRADE Working Group grades of evidence** **High certainty:** we are very confident that the true effect lies close to that of the estimate of the effect. **Moderate certainty:** we are moderately confident in the effect estimate: the true effect is likely to be close to the estimate of the effect, but there is a possibility that it is substantially different. **Low certainty:** our confidence in the effect estimate is limited: the true effect may be substantially different from the estimate of the effect. **Very low certainty:** we have very little confidence in the effect estimate: the true effect is likely to be substantially different from the estimate of effect. | | | | | | |

#### Explanations

a. Mixed surgical approach

b. Mixed type of stoma

c. Heterogeneous follow-up

d. Possible selection bias

Table 7. Summary of Lateral stoma compared to Control for Parastomal Hernia Prophylaxis
